# Mutations in the tail and rod domains of the neurofilament heavy‐chain gene increase the risk of ALS

**DOI:** 10.1002/acn3.52083

**Published:** 2024-05-22

**Authors:** Heather Marriott, Thomas P. Spargo, Ahmad Al Khleifat, Peter M Andersen, Nazli A. Başak, Johnathan Cooper‐Knock, Philippe Corcia, Philippe Couratier, Mamede de Carvalho, Vivian Drory, Marc Gotkine, John E. Landers, Russell McLaughlin, Jesús S. Mora Pardina, Karen E. Morrison, Susana Pinto, Christopher E. Shaw, Pamela J. Shaw, Vincenzo Silani, Nicola Ticozzi, Philip van Damme, Leonard H. van den Berg, Patrick Vourc'h, Markus Weber, Jan H. Veldink, Project MinE ALS Sequencing Consortium, Ahmad Al Khleifat, Ammar Al‐Chalabi, Peter M Andersen, Nazli A. Başak, Johnathan Cooper‐Knock, Philippe Corcia, Philippe Couratier, Mamede de Carvalho, Vivian Drory, Jonathan D. Glass, Marc Gotkine, Orla Hardiman, Alfredo Iacoangeli, John E. Landers, Russell McLaughlin, Jesús S. Mora Pardina, Karen E. Morrison, Susana Pinto, Monica Povedano, Christopher E. Shaw, Pamela J. Shaw, Vincenzo Silani, Nicola Ticozzi, Philip van Damme, Leonard H. van den Berg, Patrick Vourc'h, Markus Weber, Jan H. Veldink, Richard J. Dobson, Patrick Schwab, Ammar Al‐Chalabi, Alfredo Iacoangeli

**Affiliations:** ^1^ Department of Basic and Clinical Neuroscience Maurice Wohl Clinical Neuroscience Institute, Institute of Psychiatry, Psychology and Neuroscience, King's College London London SE5 8AF UK; ^2^ Department of Biostatistics and Health Informatics Institute of Psychiatry, Psychology and Neuroscience, King's College London London SE5 8AF UK; ^3^ Department of Clinical Science Umeå University Umeå SE‐901 85 Sweden; ^4^ Translational Medicine Research Center, NDAL, School of Medicine Koc University Istanbul 34450 Turkey; ^5^ Sheffield Institute for Translational Neuroscience (SITraN) University of Sheffield Sheffield S10 2HQ UK; ^6^ UMR 1253, Université de Tours, Inserm Tours 37044 France; ^7^ Centre de référence sur la SLA, CHU de Tours Tours 37044 France; ^8^ Centre de référence sur la SLA, CHRU de Limoges Limoges France; ^9^ UMR 1094, Université de Limoges, Inserm Limoges 87025 France; ^10^ Instituto de Fisiologia, Instituto de Medicina Molecular João Lobo Antunes, Faculdade de Medicina Universidade de Lisboa Lisbon 1649‐028 Portugal; ^11^ Department of Neurology Tel‐Aviv Sourasky Medical Centre Tel‐Aviv 64239 Israel; ^12^ Sackler Faculty of Medicine Tel‐Aviv University Tel‐Aviv 6997801 Israel; ^13^ Faculty of Medicine Hebrew University of Jerusalem Jerusalem 91904 Israel; ^14^ Agnes Ginges Center for Human Neurogenetics, Department of Neurology Hadassah Medical Center Jerusalem 91120 Israel; ^15^ Department of Neurology University of Massachusetts Medical School Worcester Massachusetts 01655 USA; ^16^ Complex Trait Genomics Laboratory Smurfit Institute of Genetics, Trinity College Dublin Dublin D02 PN40 Ireland; ^17^ ALS Unit Hospital San Rafael Madrid Spain; ^18^ School of Medicine, Dentistry and Biomedical Sciences Queen's University Belfast Belfast BT9 7BL UK; ^19^ Department of Neurology‐Stroke Unit and Laboratory of Neuroscience Istituto Auxologico Italiano, IRCCS Milan 20149 Italy; ^20^ Department of Pathophysiology and Transplantation, “Dino Ferrari” Center Università degli Studi di Milano Milan 20122 Italy; ^21^ Experimental Neurology and Leuven Brain Institute (LBI) Leuven 3000 Belgium; ^22^ VIB, Center for Brain and Disease Research Leuven 3000 Belgium; ^23^ Department of Neurology University Hospitals Leuven Leuven 3000 Belgium; ^24^ Department of Neurology, UMC Utrecht Brain Center University Medical Center Utrecht 3584 CX Netherlands; ^25^ Service de Biochimie et Biologie molécularie, CHU de Tours Tours 37044 France; ^26^ Neuromuscular Diseases Unit/ALS Clinic Kantonsspital St. Gallen St. Gallen 9007 Switzerland; ^27^ NIHR Biomedical Research Centre at South London and Maudsley NHS Foundation Trust and King's College London London UK; ^28^ Institute of Health Informatics, University College London London NW1 2DA UK; ^29^ NIHR Biomedical Research Centre at University College London Hospitals NHS Foundation Trust London UK; ^30^ GlaxoSmithKline, Artificial Intelligence and Machine Learning London UK; ^31^ King's College Hospital London SE5 9RS UK

## Abstract

**Objective:**

Neurofilament heavy‐chain gene (*NEFH*) variants are associated with multiple neurodegenerative diseases, however, their relationship with ALS has not been robustly explored. Still, *NEFH* is commonly included in genetic screening panels worldwide. We therefore aimed to determine if *NEFH* variants modify ALS risk.

**Methods:**

Genetic data of 11,130 people with ALS and 7,416 controls from the literature and Project MinE were analysed. We performed meta‐analyses of published case–control studies reporting *NEFH* variants, and variant analysis of *NEFH* in Project MinE whole‐genome sequencing data.

**Results:**

Fixed‐effects meta‐analysis found that rare (MAF <1%) missense variants in the tail domain of *NEFH* increase ALS risk (OR 4.55, 95% CI 2.13–9.71, *p* < 0.0001). In Project MinE, ultrarare *NEFH* variants increased ALS risk (OR 1.37 95% CI 1.14–1.63, *p* = 0.0007), with rod domain variants (mostly intronic) appearing to drive the association (OR 1.45 95% CI 1.18–1.77, *p*_Madsen–Browning_ = 0.0007, p_SKAT‐O_ = 0.003). While in the tail domain, ultrarare (MAF <0.1%) pathogenic missense variants were also associated with higher risk of ALS (OR 1.94, 95% CI 0.86–4.37, *p*_Madsen–Browning_ = 0.039), supporting the meta‐analysis results. Finally, several tail in‐frame deletions were also found to affect disease risk, however, both protective and pathogenic deletions were found in this domain, highlighting an intricate architecture that requires further investigation.

**Interpretation:**

We showed that *NEFH* tail missense and in‐frame deletion variants, and intronic rod variants are risk factors for ALS. However, they are not variants of large effect, and their functional impact needs to be clarified in further studies. Therefore, their inclusion in routine genetic screening panels should be reconsidered.

## Introduction

Amyotrophic lateral sclerosis (ALS) is a fatal neurodegenerative disease resulting from upper and lower motor neuron loss. Around 40 genes have been implicated in ALS and are involved in cellular processes such as autophagy, DNA damage repair, protein degradation, mitochondrial function, and cellular/axonal transport.^1^ The neurofilament heavy‐chain gene (*NEFH*), encodes the neurofilament heavy subunit protein (NF‐H), which regulates several of these activities to maintain neuronal homeostasis.

Neurofilament protein subunits preserve neuronal architecture by using their side‐arms to construct cross‐bridges with cytoskeletal components such as microtubules and actin filaments, forming a stable filament‐centred matrix that allows intracellular signalling, mitochondrial localisation, and ER transport to occur.^2^ This is predominantly orchestrated by the phosphorylation of the head and tail domains of neurofilament genes. For instance, phosphorylation of the head domain acts as a primer for matrix formation, controlling polymerisation of the NF‐H subunit in the cell body before the subunits move to the axon, where the lysine‐serine‐proline (KSP) repeat of the tail domain is phosphorylated to construct the matrix structure and stabilise the neurofilament side arms.^3^ As a result, *NEFH* disruption could influence selective motor neuron degeneration in the brain and spinal cord of affected individuals with ALS via dysregulation of neuronal function.^4^

Frameshift and missense mutations in *NEFH* have been convincingly linked to various neurological diseases, including Charcot–Marie–Tooth disease type 2CC,^5^, ^6^ spinal muscular atrophy,^7^ and Alzheimer's disease.^8^ Several lines of evidence suggest hyperphosphorylation of the KSP repeat causes axonal aggregation of phosphorylated NF‐H (pNF‐H), thereby compromising neuronal integrity and increasing circulating pNF‐H levels in the serum and CSF.^3^ Raised pNF‐H levels have already been established as a biomarker for ALS progression, survival,^9^, ^10^ patterns of motor neuron involvement, and can clinically distinguish ALS from mimics such as hereditary spastic paraplegia, spinal muscular atrophy, and myasthenia gravis.^11^ While pNF‐H demonstrates prognostic value, there have not been robust studies examining the relationship between *NEFH* mutations and ALS susceptibility. The association between small insertions and deletions (indels) in the KSP repeat and ALS risk has been suggestively reported in a number of studies,^12^, ^13^, ^14^ however, it has not been widely reproduced nor does solid statistical evidence exist. Still, *NEFH* is commonly included in genetic testing panels worldwide.^15^ This study aims to fill this gap by first performing a meta‐analysis of published ALS case–control studies that reported *NEFH* variants and second conducting a large‐scale investigation of *NEFH* variation using genetic data from the Project MinE international ALS whole‐genome sequencing consortium.

## Methods

### Systematic review

This study was performed in accordance with the 2020 Preferred Reporting Items for Systematic Reviews and Meta‐Analyses (PRISMA) guidelines.^16^ Registration and study protocol of the review aspect of this study was not performed.

### Eligibility criteria

Primary research articles published in the English language between January 1993 and October 2021 were included if they reported individual *NEFH* variant frequencies in ALS patients via a candidate or panel gene approach (targeted panel resequencing and variant screening), whole‐genome sequencing, whole‐exome sequencing, microarray, or PCR‐based approaches. Studies were excluded if they were clinical, functional, or epidemiological, if *NEFH* variants were not identified, were identified in non‐ALS cases only, or if individual frequencies of all variants were not reported.

### Information sources, search strategy, and screening process

Relevant studies were identified by searching PubMed, Embase, and Medline databases with the search terms “amyotrophic lateral sclerosis” OR “ALS” in combination with “neurofilament heavy chain gene,” “NEFH,” “NFH” OR “NF‐H,” After removing duplicate records, title and abstract screening was performed against the eligibility criteria, which were formatted into a table checklist. Studies which advanced to full‐text screening were subject to backwards citation screening using Web of Science to identify any articles which may have been missed. Full‐text screening of database and citation identified records was then performed. The search strategy was independently performed, and the results were crosschecked by two members of the team.

### Data collection process and data synthesis

The following characteristics were extracted from the eligible records: author, publication year, study design, screening method, and genetic technology used to detect *NEFH* variants, population (country of origin), study groups, sex, and age of ALS groups, and diagnostic criteria. For each variant, the following information was obtained: HGVS nomenclature, mutation type, *NEFH* domain location, rsID, and pathogenicity according to SIFT, PolyPhen, REVEL, and CADD. Study‐specific variant information, that is, frequency in cases and controls, odds ratios (ORs), and 95% confidence intervals with *p*‐values and other ALS‐associated gene variants carried in *NEFH*‐positive individuals, were also extracted. Population‐specific *NEFH* variant frequencies were added to each variant record using the gnomADv2.1.1 non‐neuro database.^17^ If the rsID was not supplied, dbSNP^18^ and gnomAD were searched. For variants without pathogenicity predictions, gnomAD and the Variant Effect Predictor (VEP)^19^ were used to obtain variant consequence status. All of this information was tabulated into separate study‐specific and variant‐specific characteristics tables.

### Meta‐analysis

Individual missense and exonic indel variants found in two or more case–control studies were eligible for variant‐level meta‐analysis. Subgroup meta‐analysis was also performed according to combinations of population‐specific gnomAD non‐neuro frequency (ultrarare: <0.1%, high‐frequency rare: 0.1–1%, rare: <1%, or common: >1%), domain (head, rod, or tail), and variant type. Studies that identified variants absent from gnomAD but present in more than one control were classified as common for the stratified analysis. Synonymous variants were excluded from the analysis. Inverse‐variance weighted meta‐analyses were conducted with both fixed‐effect (Cochran–Mantel–Haenszel) and random‐effect (DerSimonian–Laird) models. Crude ORs were calculated from the extracted data. Between‐study heterogeneity was assessed using the combination of the I‐squared test and Cochran‐Q statistic, with significant heterogeneity indicated when *I* ^2^ >50% and *Q* <0.10. In this case, the result from the random‐effect model is the result that we report and use. Publication bias was assessed with both Egger's and Harbord's test, with *p*‐values <0.05 classed as displaying significant outcome heterogeneity and selective reporting. All statistical analyses were performed using the *metabin* and *metabias* functions of the *meta* R package.

### Genetic screening

Whole‐genome sequencing samples collected as part of the Project MinE ALS sequencing consortium^20^ were used to investigate *NEFH* variants in ALS and for replicating the literature based meta‐analysis results. Information about recruitment and data collection is available in the Project MinE paper.^20^ In brief, samples were sequenced using PCR‐free library preparation on the Illumina HiSeq 2000 and HiSeq × platforms to ∼35× coverage with 100 bp reads and ∼25× coverage with 150 bp reads, respectively. Sequencing data alignment to GRCh37 and variant calling were performed using the Illumina Isaac pipeline. Sites with a genotype quality <10 and variants with low‐quality scores (<20 for single nucleotide variants and <30 for indels) were removed. Samples with a transition‐transversion ratio, total number of single‐nucleotide variants, indels, and singletons outside the interval mean ± 6 SD from the full distribution of samples were removed. Variants with missingness >2% across all samples were excluded. Genetically inferred sex, based on the number of X and Y chromosomes, was compared to the sex reported in the phenotypic data. The full data set consisted of 9050 individuals, 6603 ALS cases and 2,447 age‐ and sex‐matched controls. After standard quality controls, the data set comprised of 6469 ALS cases and 2434 controls from 13 countries (Supplementary Table S1) for which SNV and small indel data were available. Structural variants (SVs) generated with Manta^21^ were available for approximately two thirds of samples (4686 ALS cases and 1859 controls). Variants were annotated with VEP for both functional consequence/type (e.g. 3′UTR, 5′UTR, intronic, missense, indel, and synonymous) and impact classification (high, moderate, low, and modifier) before a union SV data set was created. SVs greater than 100,000 bp were excluded to reduce false positives. The remaining SVs were annotated with AnnotSV^22^ and CADD‐SV^23^ to assess their potential pathogenicity. All results files were converted into a matrix to calculate case–control frequencies. For the review‐identified variants and SVs present in Project MinE, Firth logistic regression was performed using RVTests^24^ with default settings, to assess potential associations between variants and ALS susceptibility. Results were corrected for sex and the first 10 principal components. All data was aligned to hg19.

### Rare variant burden analysis

Burden analysis of all *NEFH* variants identified in Project MinE was performed with RVTests, using Madsen–Browning and SKAT‐O methods with default settings. These tests were chosen as their underlying statistical approaches are different and they account for discordant directions of variant effect, which means that the likelihood of finding an association of gene burden units with ALS risk is maximised given that ALS displays a complex disease architecture. The Madsen–Browning test uses a combination of a weighted‐sum test and permutation of case status to adjust variant weights in order to identify an excess of disease‐contributing variants in a particular region, therefore this test is useful if all of the variants are causal and have similar effect sizes.^25^ On the other hand, the SKAT‐O, which adopts a variance component approach to evaluate variant distribution, considers that there will be both a large amount of variants which are not associated with the phenotype, and disease‐contributing variants which display different directions of effect, that is, causal and protective.^26^

Results were corrected for sex and the first 10 principal components. Variants were initially grouped by frequency (ultrarare: <0.1%, high‐frequency rare: 0.1–1%, rare: <1%), according to the highest value in control databases (gnomAD non‐neuro non‐Finnish European and Project MinE controls), before being grouped by functional domain (whole gene, head, rod, and tail) with the *ensembldb* R package. For each functional domain, variant burden was calculated for several variant types (missense, synonymous, insertion, deletion, 3′UTR, 5′UTR, and intronic) and VEP impact classes (high, moderate, low, and modifier). Also, the burden of missense variants predicted pathogenic by SIFT and/or PolyPhen was assessed.

### Phenotype analysis

We investigated whether the *NEFH* variants in the 18 classes highlighted in this study, for example, pathogenic tail missense and rod intronic (full list in supplementary table 14), have any effect on the ALS clinical phenotype. We performed a statistical comparison of male : female ratio, age of onset, diagnostic delay, and disease duration between people with ALS in each variant class versus those absent from each variant class. Differences in male : female ratio was assessed with the chi‐squared test. Differences in age of onset and diagnostic delay was assessed using two‐way ANOVA corrected for sex and site of onset, with differences in disease duration assessed using a Cox proportional hazards model corrected for sex and site of onset. The stats and survival R packages were used for these analyses.

### Controlling for multiple testing

In both meta‐analysis and burden analyses, we report *p*‐values <0.05 as indicators of nominal significance in addition to estimate of effects and 95% confidence intervals. In our initial discovery phase, we took multiple testing into account (Bonferroni correction) testing the association between variants and ALS risk based on four NEFH variant frequencies (MAF <0.1%, MAF 0.1–1%, MAF <1%, and MAF >1%.), that is, corrected *p*‐values <0.0125. Any additional subgroup analyses aiming to explain which types of frequency‐grouped variants contributed to the already discovered associations were considered significant if *p*‐values <0.05 and the direction of effect was concordant with the initial association.

## Results

### Study selection

**Figure 1:**
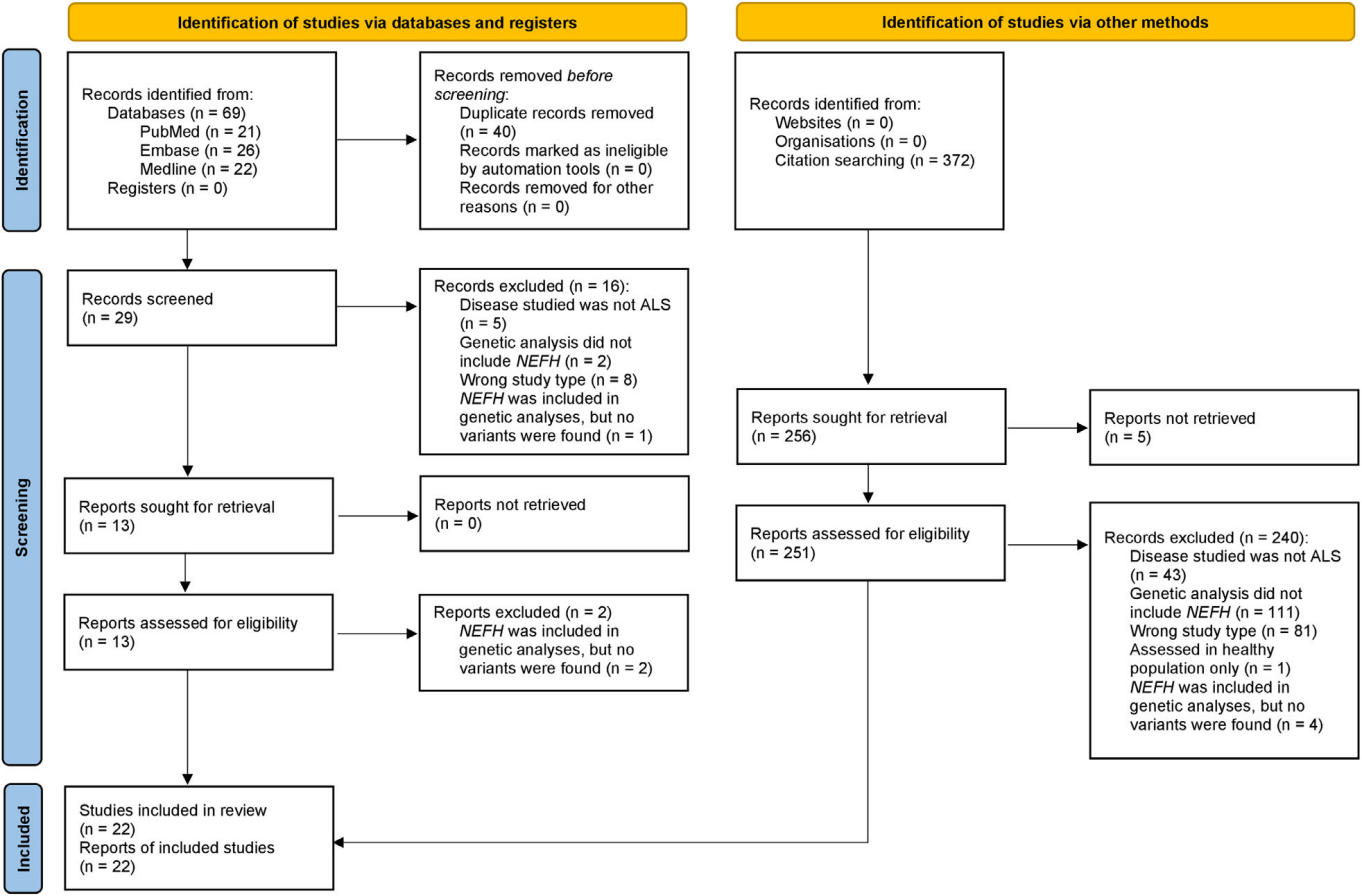
PRISMA flowchart of the study systematic review process. The left of the figure outlines screening for articles identified via PubMed, Embase, and Medline databases, while the right outlines the process for articles found via backwards citation screening of articles undergoing full‐text screening.

The systematic literature review process flowchart is presented in Figure 1. The initial search identified 29 articles which were eligible for title and abstract screening, of which 16 were the wrong study type, disease, or instances where genetic screening did not include *NEFH* or identify *NEFH* variants even if *NEFH* was present in the targeted sequencing panel. Backwards citation searching of the remaining 13 articles found an additional 251 records for screening. Manual full‐text inspection removed a further 242 records (2 from database search and 240 from citation search) as the inclusion criteria were not met. In total, 22 studies involving a total of 10,959 individuals (6090 ALS cases and 4869 controls) from 14 countries were included in the present study.

### Study characteristics

An overview of the characteristics of all included studies is given in Table S2. European (N Studies = 9) and Asian (N Studies = 7) populations were the most represented, with family disease history reported in 77% of studies. Diagnostic criteria were applied to support inclusion in 15 studies (68%), with varying definitions of El Escorial criteria employed in 93% of those. A combination of El Escorial and Awaji‐Shima criteria was used in one study. The average age of recruitment of the ALS patients ranged from 30.7 to 62.1 (median 58.1), with a male : female ratio ranging between 0.60 and 1.78 (median 1.38) across studies. When separating by country, Asian populations had a younger median age at recruitment and a higher median male : female ratio than European populations (Asian: age 52.01, sex ratio 1.52; European: age 60.1, sex ratio 1.22). A case–control design was adopted in 12 studies (55%), with 6 investigating *NEFH* variation in ALS via candidate gene‐based methods. Gene panels including *NEFH* were used in 13 studies, with a further 2 opting for custom variant panel screening. The most popular genetic technology was whole‐exome sequencing (N Studies = 6) and a combination of whole‐exome sequencing with validation approaches such as PCR and Sanger sequencing (N Studies = 6).

### Variant characteristics

**Figure 2:**
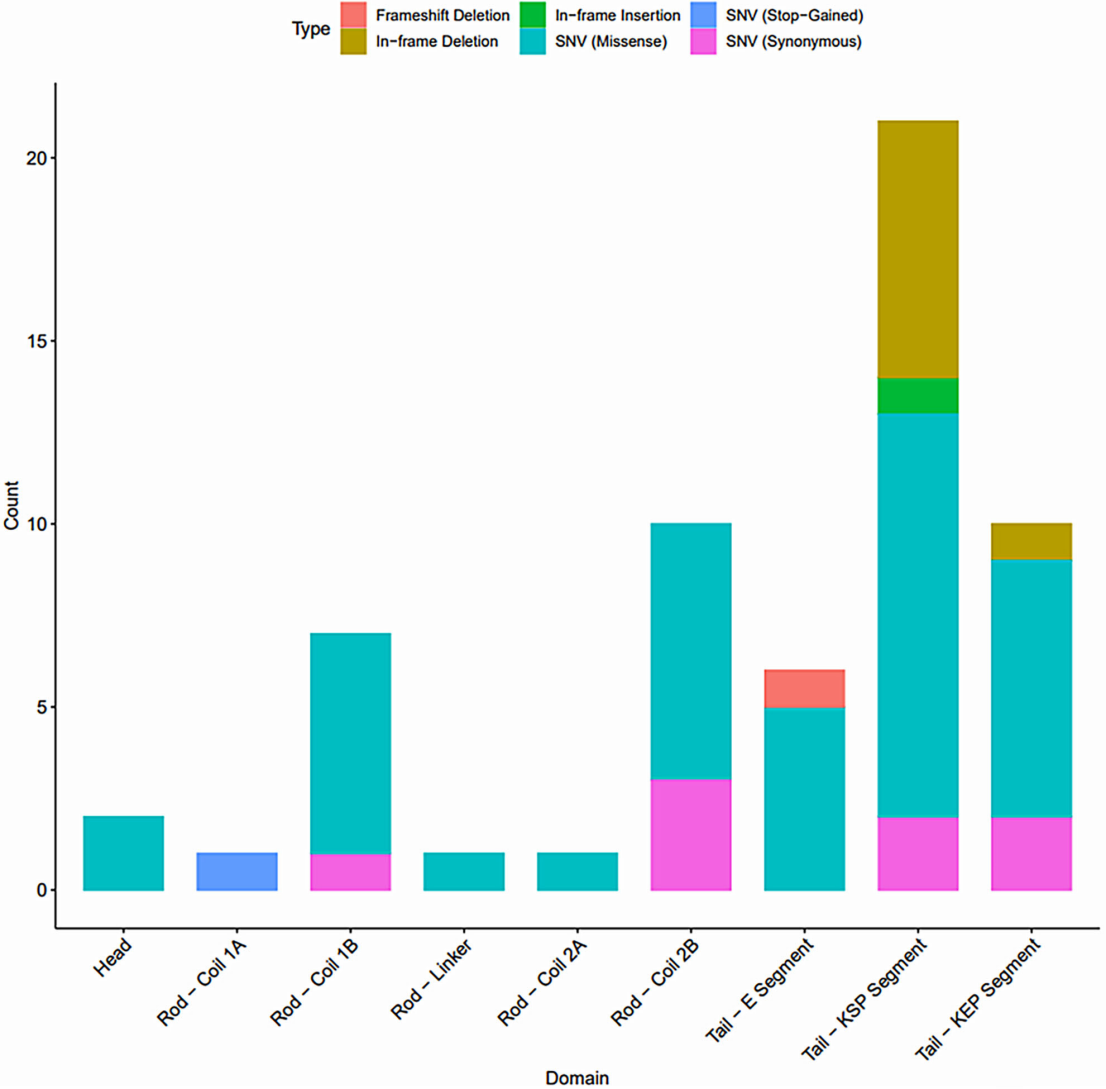
*NEFH* domain distribution of the 59 variants identified from the systematic review. Colours characterise the different variant types. KEP = lysine–glutamic acid–proline; KSP = lysine–serine–proline.

We extracted information for 59 *NEFH* variants from the included studies. The full variant details are available in Tables S3 and S4. Missense variants were the most represented (67.8%), followed by in‐frame deletions (13.6%), synonymous variants (13.6%), in‐frame insertions (1.7%), frameshift deletions (1.7%), and stop‐gained SNVs (1.7%). Indels ranged from 3 bp to 48 bp in length and exclusively occupied the tail (Fig. 2). Two variants were found in the head domain (Fig. 2). Only 18 variants (30.5%) were reported in more than one study. Eleven people with *NEFH* variants also harboured variants in other ALS‐associated genes, including *SOD1*, *FUS*, *OPTN*, *SETX*, *ALS2*, and *CHMP2B* (Table S3). When looking at the functional impact of these variants in the gnomAD database, both *OPTN* variants (M98K and R545Q) and the *ALS2* T293I variant are predicted to be tolerated and benign according to SIFT, PolyPhen, REVEL, and CADD, while the *FUS* c.37 C > T variant is predicted as benign with CADD and SpliceAI. The remaining variants have conflicting pathogenicity results with SIFT, PolyPhen, REVEL, and CADD, therefore their influence on ALS risk in individuals harbouring these variants are uncertain.

### Meta‐analysis of previously published studies

**Figure 3:**
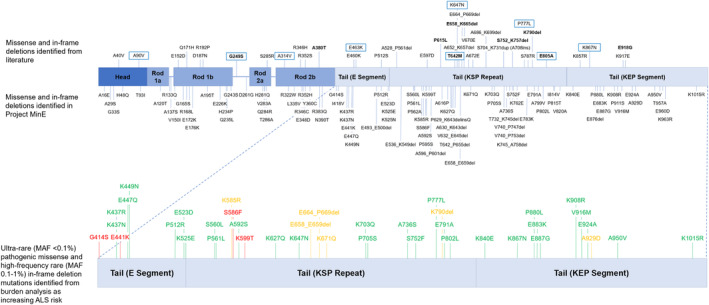
Schematic depicting the locations of the gene variants included in the meta‐analysis and in both meta‐analysis and Project MinE (top), as well as the variants that were found to increase the risk for ALS with burden analysis (bottom). Green = only present in cases. Amber = present in cases and controls. Red = only present in controls.

Twelve case–control studies were selected for meta‐analysis. In total, 34 deletion, insertion, and missense variants were reported across them (top panel of Fig. 3) in 9496 individuals (4527 cases; 4969 controls). Of these, 9 variants (3 in‐frame deletions and 6 missense) were identified in two or more case–control studies and were included in the variant‐level meta‐analysis. No singular variant was shown to significantly alter risk for ALS (Table S5), with K790del displaying high between‐study heterogeneity (Cochran's *Q* = 3.03, *p* = 0.08; *I* ^2^ = 67%).

**Figure 4:**
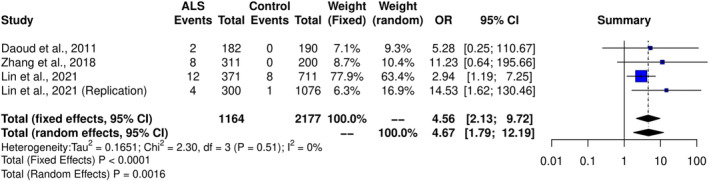
Forest plot demonstrating that rare (MAF <1%) missense variants in the tail domain increase the risk of ALS. Breakdown of heterogeneity values are as follows: *I* ^2^ = *I* ^2^ statistic, *τ* ^2^ = tau‐squared (estimate of between‐study variance in random‐effects models), χ⅔ = Cochrane's Q (chi‐squared distribution).

We then performed meta‐analyses of *NEFH* variants based on the aggregation of variants stratified by frequency, domain, and variant type (Table S6). We found that both rare missense and rare tail variants were associated with an increased risk of ALS (Table S6). We determined that the rare missense tail variants were driving this result as they were more significant, with a higher OR (OR 4.55, 95% CI 2.13–9.71, *p*_fixed‐effect_ <0.0001, Figure 4), and removing them from the rare missense and rare tail meta‐analyses caused these associations to be lost. There was no evidence of inter‐study heterogeneity (Cochran's *Q* = 2.30, *p* = 0.51; *I* ^2^ = 0%) or publication bias (Egger t = 2.07, *p* = 0.17; Harbord t = 1.82, *p* = 0.21). By further stratifying *NEFH* variants into high‐frequency rare and ultrarare categories, we identified a similar but albeit weaker significant association for the high‐frequency rare missense tail variants (OR 3.91, 95% CI 1.77–8.64, *p*_fixed‐effect_ = 0.0007), with ultrarare missense tail variants approaching significance for increasing ALS risk and a showing consistent effect (OR 5.05, 95% CI 0.84–30.22; *p*_fixed‐effect_ = 0.08). Across all categories, tail deletions did not significantly increase or reduce susceptibility for ALS.

### Screening of *NEFH* SNVs and indels in the Project MinE cohort

**Figure 5:**
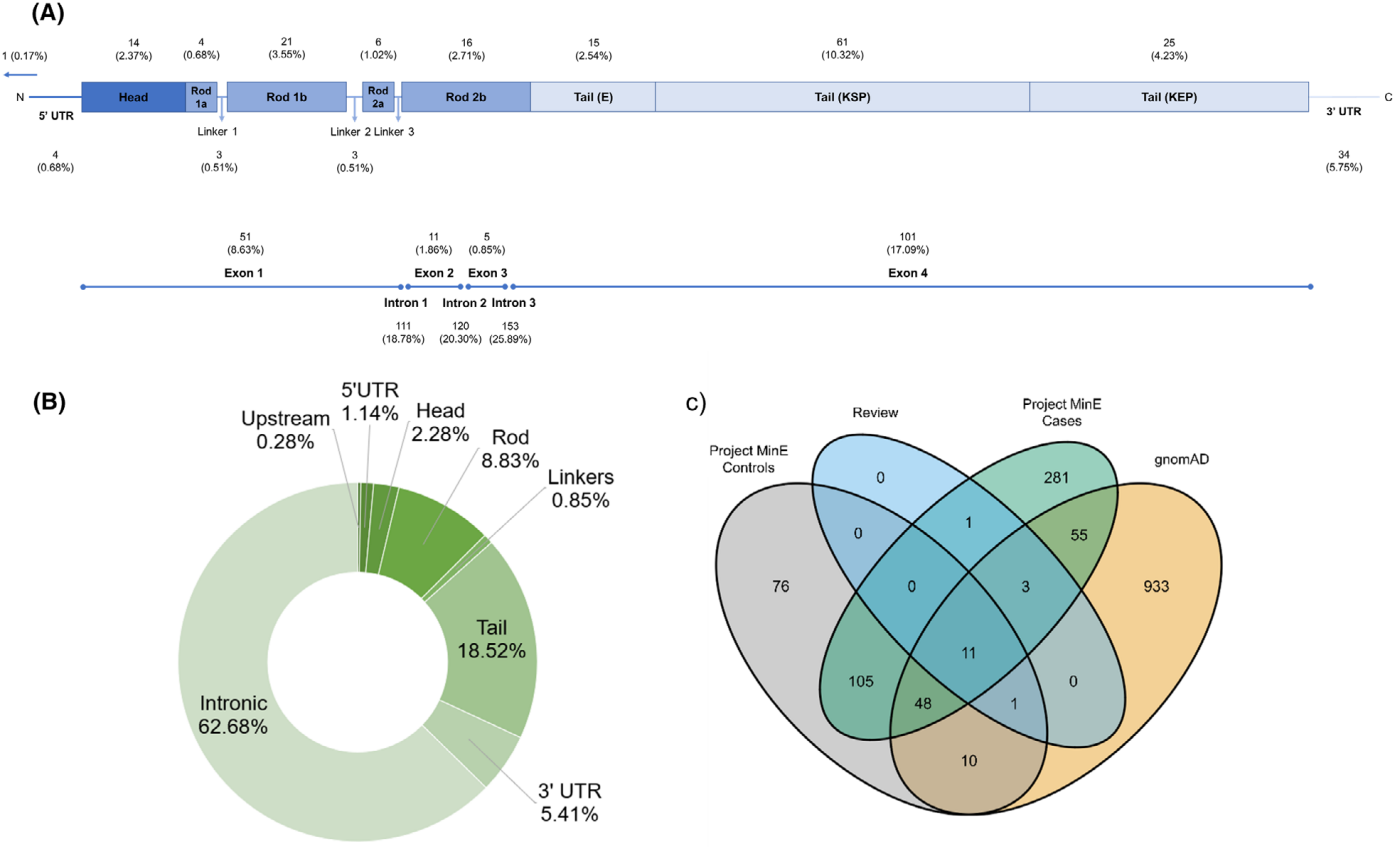
Results of the SNV/indel screening analysis in the Project MinE cohort. Additional information on all 591 variants identified are available in Table S7. (A) Proportion of variants found in various gene domains and untranslated regions (top), and in exons and introns (bottom). (B) Breakdown of the 351 *NEFH* singletons by domain. (C) A Venn diagram illustrating the overlap of the *NEFH* variants in Project MinE cases and controls, the systematic review and the gnomAD v2.1.1 database. The value for variants that are only in gnomAD (933) refers to the remaining *NEFH* variants in the catalogue after accounting for variants shared with Project MinE or the review.

We screened the whole *NEFH* gene in the Project MinE data set (6469 ALS cases and 2434 controls). A total of 591 SNVs and indels were identified (Fig. 5A and Table S7). Interestingly, intronic regions contained 65% of all variants found in the cohort, with 220 (57.29%) being singletons (Fig. 5B). In total, 462 (78.17%) were identified only in Project MinE and not in the review or gnomAD non‐neuro non‐Finnish database (Fig. 5C) and are therefore classified as “novel” in this study.

Sixteen (27.1%) of the *NEFH* variants identified from the systematic review were found in Project MinE (Fig. 5C). Examination of case–control frequencies of review‐identified variants present in Project MinE (Table S8) suggested that K790del could be protective against ALS (0.14% cases, 0.37% controls; OR = 0.38, 95% 0.15–0.95, *p* = 0.03). Using Project MinE as an additional study for meta‐analysis of individual review‐identified variants did not offer any additional insight into their role in ALS risk (Table S9).

### Screening of *NEFH* structural variants in the Project MinE cohort

Only 4 SVs were identified in Project MinE (Table S8). All were in the KSP and KEP segments of the tail domain, and none were pathogenic according to CADD‐SV. When comparing case–control frequencies of the SVs, the 113 bp KEP segment deletion was found to be strongly protective against ALS (17.95% cases vs. 23.91% controls; OR 0.72, 95% CI 0.64–0.81, *p* = 2.6E‐08).

### Rare variant burden analysis in the Project MinE cohort

All the SNV/indel variants from Project MinE were subject to burden analysis stratified by frequency and domain (Tables S10–S12). We found that ultrarare variants in *NEFH* increase ALS risk (OR 1.37 95% CI 1.14–1.63, Madsen–Browning *p* = 0.0007, SKAT‐O *p* = 0.0033). When stratifying by domain and functional effect, ultrarare variants, mostly intronic, in the rod domain appeared to drive the association (OR 1.45 95% CI 1.18–1.77, Madsen–Browning *p* = 0.0007, SKAT‐O *p* = 0.003). However, ultrarare pathogenic missense tail variants were also associated with an increased risk of ALS (OR 1.94, 95% CI 0.86–4.37; Madsen–Browning *p* = 0.039), which supported the result of the meta‐analysis to a high degree, despite high‐frequency rare pathogenic missense tail variants in this cohort not appearing to confer ALS risk (Table S11). Stratifying this by subdomain revealed that the KEP repeat drove this result (OR 5.65, 95% CI 0.75–42.83, Madsen–Browning *p* = 0.02), and that other sub‐domains showed consistent, although not significant, effects (OR >1) (Table S10). In line with previous reports, ultrarare tail domain in‐frame deletions had a large impact on ALS risk, but this finding was at the border of the significance testing threshold (OR 3.01, 95% CI 0.69–13.12, Madsen–Browning *p* = 0.052). A similar but significant effect was observed for high‐frequency rare in‐frame deletions, with an OR of 1.18 (95% CI 0.67–2.07, SKAT‐O *p* = 0.03). Ultrarare pathogenic missense variants and high‐frequency rare in‐frame deletions identified and assessed in these burden analyses are detailed in Figure 3 (bottom panel) and Table S13.

### Phenotype analysis in project MinE

Finally, we investigated whether the variants found to affect the risk of ALS in our study, are associated with different clinical outcomes (age of onset, survival, and diagnostic delay) using the clinical data available in Project MinE and stratifying the variants in 18 groups according to functional impact, domain and MAF (Table S14). Overall, patients with mutations in the *NEFH* gene had an older age of onset (mean difference 1.28 years ±0.34 years, *p* = 2.12E‐04), and carrying the 113 bp KEP segment deletion was associated with an even older age of onset (mean difference 2.87 years ±0.42 years, *p* = 8.66E‐12). No other test yielded a significant difference after Bonferroni multiple testing correction (18 tests per clinical outcome). Complete results are available in Table S14.

## Discussion

Leveraging genetic data from 11,130 people with ALS and 7416 controls from both the literature and Project MinE, we found that rare variants in the *NEFH* gene increase the risk of ALS (Project MinE *p* = 0.0007 and meta‐analysis *p* < 0.0001) and that this association was driven by missense variants in the tail domain and intronic variants in the rod domain. Our meta‐analysis reported that rare (MAF <1%) missense tail *NEFH* variants in a total of 1164 people with ALS and 2177 controls yielded an OR of 4.55 (*p* < 0.0001). This was replicated to some extent, although with a lower OR, when performing ultrarare variant burden analysis of pathogenic missense tail variants in the Project MinE data set (OR 1.94, Madsen–Browning *p* = 0.039). The difference between effect sizes is likely due to the discrepancy in sample size between the two cohorts, with smaller sample sizes often reporting a larger effect size for significant relationships in either direction^27^, or also the “winner's curse” effect commonly observed in genetic association discovery studies.^28^ These findings hold high validity as most missense variants in the meta‐analysis are deleterious and possibly/probably damaging according to SIFT and PolyPhen (Table S3), which are the same criteria used in the Project MinE burden analyses.

The effect of missense tail variants on *NEFH* is not clear,^4^ but it is plausible to hypothesise that they could modify the effects of phosphorylation, thereby changing the conformation of the NF‐H subunit in such a way that simultaneously increases the propensity of pNF‐H aggregate formation in the axon and disrupts energy metabolism and protein transport. Therefore, future studies should incorporate genetic evidence of missense tail mutations with proteomic and transcriptomic data to determine if the aberrant stoichiometry of NF‐H is due solely to the action of genetic mutations or is a product of a more complex interaction between miRNA and protein targets.

We also found that rare small in‐frame deletions in the tail domain confer susceptibility to ALS within Project MinE, which agrees with previous findings in the literature,^12^, ^13^ but not in the meta‐analysis. This discrepancy could again be due to the relatively small sample sizes used in the meta‐analysis compared to Project MinE, or that there may be subdomain‐specific effects occurring in the tail that the meta‐analysis design could not account for. Potentially, deletions in the KSP repeat could be associated with an increased risk for ALS and that perhaps deletions in the KEP segment may dilute this association, having a protective effect. This is plausible given that we found a novel protective 113 bp deletion in this region, and recent studies identified other protective variants in the gene.^29^, ^30^

An interesting finding was that ultrarare (MAF <0.1%) intronic rod SNVs and indels were the main drivers of the association between *NEFH* variants and ALS (Table S10). A recent study reported a noncoding repeat polymorphism in the same domain as being protective against ALS^30^, suggesting an intricate genetic architecture in this domain with different classes of variants playing different roles. In recent years, several studies have shown that noncoding variants have a major role in ALS: for example, enhancer variants in *CAV1* and *CAV2* ^31^, ^32^ and intronic variants in *UNC13A*.^33^, ^34^ Our findings expand this by also implicating *NEFH* noncoding variants in ALS. However, understanding the functional effect of variants in noncoding regions is challenging and further research is needed to understand how noncoding neurofilament variants contribute to ALS risk. Finally, our investigation of the effect of *NEFH* variants on the ALS phenotype suggest that patients carrying mutations in the *NEFH* gene have a mean age of onset approximately 1.3 years older than other patients. However, this result was not consistent across all *NEFH* variants and was likely driven by the 113 bp KEP segment deletion whose carriers presented a mean age of onset almost 3 years older.

In conclusion, we demonstrated that missense variants and in‐frame deletions in the tail domain, and intronic variants in the rod domain of *NEFH* are associated with an increased risk of ALS. However, their functional impact needs to be assessed in further experimental studies, and because they are not variants of large effect their inclusion in routine genetic screening panels should be reconsidered as they hold limited value for genetic counselling.

## Author Contributions

HM, AA‐C, and AI contributed to conception and design of the study; HM, TPS, AAK, and AI contributed to the acquisition and analysis of data; all authors contributed to data collection and generation; all authors contributed to writing the manuscript or preparing the figures.

## Conflict of Interest

H.M. is supported by GlaxoSmithKline. T.P.S is employed by AstraZeneca. A.A‐C reports consultancies or advisory boards for Amylyx, Apellis, Biogen, Brainstorm, Cytokinetics, GenieUs, GSK, Lilly, Mitsubishi Tanabe Pharma, Novartis, OrionPharma, Quralis, Sano, Sanofi, and Wave Pharmaceuticals. The other authors have nothing to report.

## Supporting information


Table S1.

## Data Availability

All data relevant to meta‐analysis are included in the article or uploaded as supplementary information. For screening analysis, the genetic data are available upon reasonable request via Project MinE (www.projectmine.com).
