# Supplementary material for: Mutations in the tail and rod domains of the neurofilament heavy‐chain gene increase the risk of ALS
Source: Ann Clin Transl Neurol. 2024 May 22;11(7):1775–86. doi: 10.1002/acn3.52083 (PMC11251467; doi:10.1002/acn3.52083)
Supplement: Supplementary file 1 — Table S1. [file ACN3-11-1775-s001.docx]

**Mutations in the tail and rod domains of the neurofilament heavy chain gene increase the risk of ALS**

Heather Marriott MSc^1,2^, Thomas P. Spargo MSc^1,2^, Ahmad Al Khleifat PhD^1^, Peter M Andersen MD, PhD^3^, Nazli A. Başak PhD^4^, Johnathan Cooper-Knock PhD^5^, Philippe Corcia MD, PhD^6,7^, Philippe Couratier^8,9^, Mamede de Carvalho^10^, Vivian Drory MD^11,12^, Marc Gotkine MD^13,14^, John E. Landers PhD^15^, Russell McLaughlin PhD^16^, Jesús S. Mora Pardina^17^, Karen E. Morrison^18^, Susana Pinto MD, PhD^10^, Christopher E. Shaw MD^1^, Pamela J. Shaw MD^5^, Vincenzo Silani MD^19,20^, Nicola Ticozzi MD^19,20^, Philip van Damme MD, PhD^21,22^, Leonard H. van den Berg MD, PhD^23^, Patrick Vourc’h PhD^6,24^, Markus Weber PhD^25^, Jan H. Veldink PhD^23^, Project MinE ALS Sequencing Consortium, Richard J. Dobson PhD^2,26,27,28^, Patrick Schwab PhD^29^, Ammar Al-Chalabi MD, PhD^1,30*^, Alfredo Iacoangeli PhD^1,2,26*,**^

^1^Maurice Wohl Clinical Neuroscience Institute, Department of Basic and Clinical Neuroscience, Institute of Psychiatry, Psychology and Neuroscience, King’s College London, London, SE5 8AF, UK

^2^Department of Biostatistics and Health Informatics, Institute of Psychiatry, Psychology and Neuroscience, King’s College London, London, SE5 8AF UK

^3^Department of Clinical Science, Umeå University, Umeå SE-901 85, Sweden

^4^Koc University, School of Medicine, Translational Medicine Research Center, NDAL, Istanbul, 34450, Turkey

^5^Sheffield Institute for Translational Neuroscience (SITraN), University of Sheffield, Sheffield S10 2HQ, UK

^6^UMR 1253, Université de Tours, Inserm, Tours 37044, France

^7^Centre de référence sur la SLA, CHU de Tours, Tours 37044, France

^8^Centre de référence sur la SLA, CHRU de Limoges, Limoges, France

^9^UMR 1094, Université de Limoges, Inserm, Limoges 87025, France

^10^Instituto de Fisiologia, Instituto de Medicina Molecular João Lobo Antunes, Faculdade de Medicina, Universidade de Lisboa, Lisbon 1649-028, Portugal

^11^Department of Neurology, Tel-Aviv Sourasky Medical Centre, Tel-Aviv 64239, Israel

^12^Sackler Faculty of Medicine, Tel-Aviv University, Tel-Aviv 6997801, Israel

^13^Faculty of Medicine, Hebrew University of Jerusalem, Jerusalem 91904, Israel

^14^Agnes Ginges Center for Human Neurogenetics, Department of Neurology, Hadassah Medical Center, Jerusalem 91120, Israel

^15^Department of Neurology, University of Massachusetts Medical School, Worcester, MA 01655, USA

^16^Complex Trait Genomics Laboratory, Smurfit Institute of Genetics, Trinity College Dublin, Dublin D02 PN40, Ireland

^17^ALS Unit, Hospital San Rafael, Madrid, Spain

^18^School of Medicine, Dentistry and Biomedical Sciences, Queen's University Belfast, Belfast BT9 7BL, UK

^19^Department of Neurology-Stroke Unit and Laboratory of Neuroscience, Istituto Auxologico Italiano, IRCCS, Milan 20149, Italy

^20^Department of Pathophysiology and Transplantation, "Dino Ferrari" Center, Università degli Studi di Milano, Milan 20122

^21^Experimental Neurology and Leuven Brain Institute (LBI), Leuven 3000, Belgium

^22^VIB, Center for Brain and Disease Research, Leuven 3000, Belgium & University Hospitals Leuven, Department of Neurology, Leuven 3000, Belgium

^23^Department of Neurology, UMC Utrecht Brain Center, University Medical Center Utrecht 3584 CX, Netherlands

^24^Service de Biochimie et Biologie molécularie, CHU de Tours, Tours 37044, France

^25^Neuromuscular Diseases Unit/ALS Clinic, Kantonsspital St. Gallen, 9007 St. Gallen, Switzerland

^26^NIHR Biomedical Research Centre at South London and Maudsley NHS Foundation Trust and King’s College London, UK

^27^Institute of Health Informatics, University College London, London, NW1 2DA, UK

^28^NIHR Biomedical Research Centre at University College London Hospitals NHS Foundation Trust, London, UK

^29^GlaxoSmithKline, Artificial Intelligence and Machine Learning

^30^King’s College Hospital, London, SE5 9RS, UK

* these authors contributed equally, ** corresponding author

Corresponding author: Alfredo Iacoangeli

Corresponding author’s address: Room C0.29 ground floor, Social, Genetic and Developmental Psychiatry Centre (MRC), Institute of Psychiatry, Psychology and Neuroscience, De Crespigny Park, Denmark Hill, London, United Kingdom, SE5 8AF

Corresponding author’s phone and fax: 0044 (0)7434885640

Corresponding author’s e-mail address: [alfredo.iacoangeli@kcl.ac.uk](mailto:alfredo.iacoangeli@kcl.ac.uk)

Contents

Supplementary Table 14

Supplementary Table 25

Supplementary Table 38

Supplementary Table 413

Supplementary Table 516

Supplementary Table 617

Supplementary Table 720

Supplementary Table 854

Supplementary Table 956

Supplementary Table 1058

Supplementary Table 1159

Supplementary Table 1260

Supplementary Table 1361

Supplementary Table 1465

References67

| **Country** | **Cases** | **Controls** | **Total** |
| --- | --- | --- | --- |
| Belgium | 561 | 89 | 650 |
| Netherlands | 1854 | 1081 | 2935 |
| France | 251 | 40 | 291 |
| Italy | 55 | 0 | 55 |
| US | 435 | 80 | 515 |
| UK | 1445 | 453 | 1898 |
| Turkey | 618 | 150 | 768 |
| Ireland | 467 | 234 | 701 |
| Israel | 105 | 0 | 105 |
| Portugal | 53 | 19 | 72 |
| Sweden | 204 | 112 | 316 |
| Switzerland | 44 | 1 | 45 |
| Spain | 377 | 175 | 552 |
| **Total** | **6469** | **2434** | **8903** |

Supplementary Table 1. Breakdown of Project MinE samples used for variant screening and burden analysis by country of sample collection.

| **Reference** | **Study Type** | **Discovery Method** | **Genetic Technology** | **Population (Country)** | **Sample Groups** | **Sex (M:F (Ratio))** | **Age (years (SD or range))** | **Diagnostic Criteria** |
| --- | --- | --- | --- | --- | --- | --- | --- | --- |
| (1) | Case-Control | Candidate Gene/Region | PCR | France and America | Case: 356 SALS Control: 306 neurologically healthy unrelated individuals | - | - | Definite ALS (El Escorial) |
| (2) | Case-Control | Candidate Gene/Region | PCR-SSCP | - | Case: 100 FALS + 75 SALS Control: 100 unrelated individuals | - | - | - |
| (3) | Case-Control | Candidate Gene/Region | PCR | UK | Case: 164 ALS Control: 207 age-matched unrelated individuals | - | - | - |
| (4) | Case-Control | Candidate Gene/Region | PCR-SSCP | UK and Scandinavia (Denmark, Norway, Sweden, and Finland) | Case: UK: 19 FALS + 188 SALS Scandinavia: 59 FALS + 264 SALS Control: UK: 219 age, sex, and ethnicity-matched individuals Scandinavia: 228 age, sex, and ethnicity-matched individuals | UK SALS: 109:79 (1.38) Scandinavian Cases: 194:129 (1.50) | UK SALS: 55.2 Scandinavian Cases: 61.5 | - |
| (5) | Case-Control | Candidate Gene/Region | PCR | America | Case: 100 FALS + 100 SALS Control: 100 neurologically healthy individuals | - | - | - |
| (6) | Case-Control | Gene Panel | PCR | France and Canada | Case: 80 FALS + 110 SALS Control: 190 neurologically healthy individuals | 104:86 (1.21) | 55.4 ± 13.1 | Definite or probable ALS (El Escorial) |
| (7) | Case-Control | Gene Panel | WES | America | Case: 242 SALS Control: 29 age-matched individuals | 131:111 (1.18) | 60 (44-82) | Definite, probable, or possible ALS (El Escorial) |
| (8) | Case-Control | Gene Panel | WES + PCR | Japan | Case: 39 FALS + 469 SALS Control: 191 neurologically healthy individuals | 298:210 (1.42) | 62.1 (53.5-68.4) | Definite, probable, probable laboratory-supported or possible ALS (El Escorial) |
| (9) | Case | Gene Panel | NGS | Germany | Case: 80 ALS | 44:36 (1.22) | 60.1 (29-88) | - |
| (10) | Case-Control | Gene Panel | WGS + WES | China (Hong-Kong) | Case: 8 FALS + 46 SALS Control: 699 volunteer individuals | FALS: 3:5 (0.60) SALS: 28:18 (1.56) | FALS: 41.4 ± 8.71 SALS: 58.1 ± 13.45 | Definite, probable, or probable laboratory-supported ALS (El Escorial) |
| (11) | Case-Control | Gene Panel | WES + PCR | UK | Case: 131 FALS + 995 SALS Control: 613 neurologically healthy age and ethnicity-matched individuals | FALS: 67:64 (1.05) SALS: 567:428 (1.32) | FALS: 56 (24-85) SALS: 61 (25-88) | - |
| (12) | Case | Gene Panel | WES + Sanger | Japan | 51 FALS | - | - | El Escorial |
| (13) | Case | Variant Panel | WES | Australia | 120 ALS | 75:45 (1.67) | 61 ± 10.1 | Definite or probable ALS (revised El Escorial) |
| (14) | Case | Gene Panel | WES + PCR | Germany | 171 FALS | - | - | El Escorial |
| (15) | Case-Control | Gene Panel | WES + Sanger | China | Case: 311 SALS Control: 200 neurologically healthy individuals | 199:112 (1.78) | 51.92 ± 10.8 | Definite, probable, probable laboratory-supported or possible ALS (El Escorial) |
| (16) | Case | Gene Panel | WES | China | 24 FALS + 21 early-onset SALS | FALS: 13:11 (1.18) SALS: 13:8 (1.63) | FALS: 40.3 ± 14.8 SALS: 30.7 ± 11.5 | El Escorial |
| (17) | Case | Gene Panel | WES | Hungary | 107 ALS | 45:62 (0.73) | 60 (30-79) | El Escorial + Awaji-Shima |
| (18) | Case | Gene Panel | WES + Sanger | China | 268 ALS | 160:108 (1.48) | 52.1 ± 10.4 | Definite or probable ALS (El Escorial) |
| (19) | Case-Control | Candidate Gene/Region | PCR | China | Case: 671 SALS Control: 1787 neurologically healthy individuals | 410:261 (1.57) | 53.45 ± 9.96 | El Escorial |
| (20) | Case Report | Whole Genome | WES | Martinique | 5 related FALS | - | - | - |
| (21) | Case | Gene Panel | WES | UK | 7 FALS + 93 SALS | 54:46 (1.17) | 60.4 (22-87) | Definite, probable, probable laboratory-supported or possible ALS (revised El Escorial) |
| (22) | Case | Variant Panel | WGS | Australia | 616 SALS | 346:221 (1.57) | 60 ± 12 | Definite or probable ALS (El Escorial) |

Supplementary Table 2. Summary characteristics of all included studies identified from the systematic review. All case-control studies (12) proceeded to the meta-analysis stage. FALS = familial ALS, SALS = sporadic ALS, WES = whole exome sequencing, WGS = whole genome sequencing, PCR = polymerase chain reaction, NGS = next-generation sequencing, PCR-SSCP = polymerase chain reaction-single-strand conformation polymorphism.

| **Variant Name (HGVS Protein Notation)** | **Domain** | **rsID** | **SIFT Prediction** | **PolyPhen Prediction** | **REVEL Prediction** | **CADD Prediction** | **Case Frequency** | **Control Frequency** | **gnomAD v2.1.1 non-neuro Frequency** | **Other Carried Variants** | **Statistical Analysis Results** | **References** |
| --- | --- | --- | --- | --- | --- | --- | --- | --- | --- | --- | --- | --- |
| E459Gfs*7 | Tail (E) | rs59297913 | Deleterious | Benign | - | 32.0 | 1/171 | - | N/A | - | - | (14) |
| A528_P561del | Tail (KSP) | rs2063061681 | - | - | - | - | 1/356 | 0/306 | N/A | - | - | (1) |
| A652_K657del | Tail (KSP) | rs147489453 | - | - | - | - | 55/371 | 106/711 | 0.8726 | - | OR 0.98 (95% CI 0.77-1.26); p=0.89 | (19) |
| E658_K665del | Tail (KSP) | rs1183993443 | - | - | - | 12.9 | 1/207 | 0/219 | 0.01947 | - | - | (4) |
|  |  |  |  |  |  |  | 2/371 | 6/711 | 0.02722 | - | OR 0.64 (95% CI 0.13-3.17); p=0.72 | (19) |
| E664_P669del | Tail (KSP) | rs1317619836 | - | - | - | 14.1 | 1/207 | 1/219 | 0.0005205 | - | - | (4) |
| A686_K699del | Tail (KSP) | - | - | - | - | - | 2/323 | 1/228 | N/A | - | - | (4) |
| S752_K757del | Tail (KSP) | rs570663492 | - | - | - | 10.6 | 1/323 | 2/228 | 0.0003574 | - | - | (4) |
|  |  |  |  |  |  |  | 1/616 | - |  | OPTN M98K | - | (22) |
|  |  |  |  |  |  |  | 56/371 | 102/711 | 0.1138 |  | OR 1.07 (95% CI 0.83-1.37); p=0.61 | (19) |
| K790del | Tail (KSP) | rs59551486 | - | - | - | 10.6 | 4/356 | 0/306 | 0.001226 | - | - | (1) |
|  |  |  |  |  |  |  | 1/1126 | 2/613 |  | - | - | (11) |
| K857del | Tail (KEP) | rs762969612 | - | - | - | 9.79 | 1/171 | - | 0.00002324 | - | - | (14) |
| S704_K731dup  (A708ins) | Tail (KSP) | - | - | - | - | - | 1/164 | 0/209 | N/A | - | - | (3) |
| A40V | Head | rs1474351396 | Tolerated | Benign | 0.166 | 14.7 | 1/185 | 1/190 | 0 | - | - | (6) |
| A90V | Head | rs61556467 | Tolerated | Benign | 0.127 | 19.9 | 1/100 | 0/100 | 0.0005063 | - | - | (5) |
|  |  |  |  |  |  |  | 1/185 | - |  | - | - | (6) |
| R148P | Rod (Coil 1B) | - | Tolerated | Possibly Damaging | 0.571 | 22.2 | 1/107 | - | N/A | - | - | (17) |
| E152D | Rod (Coil 1B) | rs774792100 | Tolerated | Benign | 0.338 | 22.2 | 3/268 | - | 0.003991 | - | - | (18) |
|  |  |  |  |  |  |  | 1/371 | 1/711 |  | - | OR 1.92 (95% CI 0.12-30.70); p=0.76 | (19) |
| Q171H | Rod (Coil 1B) | - | Tolerated | Benign | 0.234 | 13.35 | 1/371 | 0/711 | N/A | - | OR Inf (95% CI Na-Inf); p=0.34 | (19) |
| D187N | Rod (Coil 1B) | rs1372062630 | Deleterious | Probably Damaging | 0.512 | 23.7 | 1/469 | 0/191 | 0 | SOD1 L39V | - | (8) |
|  |  |  |  |  |  |  | 1/51 | - | 0 | - | - | (12) |
| R192P | Rod (Coil 1B) | rs2063001424 | Deleterious | Possibly Damaging | 0.556 | 23.5 | 1/242 | 0/129 | N/A | - | - | (7) |
| A203P | Rod (Coil 1B) | - | Tolerated | Possibly Damaging | 0.378 | 14.94 | 1/51 | - | N/A | SETX C1554G | - | (12) |
| G249S | Rod (linker between Coil 1B and 2A) | rs60825978 | Tolerated | Benign | 0.195 | 12.9 | 1/100 | 0/100 | 0.007444 | - | - | (5) |
|  |  |  |  |  |  |  | 1/268 | - | 0.0305 | - | - | (18) |
|  |  |  |  |  |  |  | 3/371 | 8/711 |  | - | - | (19) |
| S285R | Rod (Coil 2A) | rs1479105421 | Deleterious | Benign | 0.427 | 23.2 | 1/469 | 0/191 | 0.0005071 | - | - | (8) |
|  |  |  |  |  |  |  | 1/51 | - |  | - | - | (12) |
| A314V | Rod (Coil 2B) | rs539511579 | Deleterious | Probably Damaging | 0.583 | 24.9 | 1/371 | 0/711 | 0.0002238 | - | OR Inf (95% CI Na-Inf); p=0.34 | (19) |
| T338I | Rod (Coil 2B) | rs774252076 | Deleterious | Damaging | 0.818 | 25.3 | 2/107 | - | 0.00003355 | - | - | (17) |
| R346H | Rod (Coil 2B) | rs1401155915 | Tolerated | Possibly Damaging | 0.635 | 27.8 | 1/186 | 0/190 | 0 | - | - | (6) |
| R352S | Rod (Coil 2B) | rs149955255 | Deleterious | Possibly Damaging | 0.713 | 24.8 | 2/200 | 2/100 | 0.003516 | - | - | (5) |
| A380T | Rod (Coil 2B) | rs201416955 | Tolerated | Possibly Damaging | 0.475 | 23.9 | 1/100 | 0/100 | 0.00001939 | - | - | (5) |
|  |  |  |  |  |  |  | 2/311 | 0/200 | 0.005275 | OPTN R545Q | - | (15) |
|  |  |  |  |  |  |  | 2/268 | - |  | - | - | (18) |
|  |  |  |  |  |  |  | 3/371 | 8/711 |  | - | OR 0.72 (95% CI 0.19-2.71); p=0.76 | (19) |
| A400V | Rod (Coil 2B) | rs757021413 | Deleterious | Probably Damaging | 0.763 | 27.2 | 51/190 | - | N/A | - | - | (6) |
| R412Q | Rod (Coil 2B) | rs763701610 | Deleterious | Possibly Damaging | 0.754 | 24.7 | 1/51 | - | 0.00005584 | ITPR2 A612T, FUS c.-37C>T, ALS2 I1373M | - | (23) |
| E460K | Tail (E) | - | - | - | - | - | 20/100 | 8/100 | N/A | - | - | (2) |
| E463K | Tail (E) | rs59371099 | Deleterious | Probably Damaging | 0.527 | 26.3 | 32/180 | - | 0.09682 | - | - | (6) |
|  |  |  |  |  |  |  | 173/1126 | 109/613 |  | - | - | (11) |
| E491K | Tail (E) | - | Tolerated | Possibly Damaging | 0.297 | 22.6 | 1/268 | - | N/A | - | - | (18) |
| P505L | Tail (E) | rs1414968372 | Deleterious | Benign | 0.256 | 8.47 | 1/107 | - | N/A | GRN C335R | - | (17) |
| P512S | Tail (E) | - | Deleterious | Possibly Damaging | 0.299 | 25.0 | 1/371 | 0/711 | N/A | - | OR Inf (95% CI Na-Inf); p=0.34 | (19) |
| E597D | Tail (KSP) | rs753850528 | Tolerated | Benign | 0.054 | 0.064 | 1/311 | 0/200 | 0.0002236 | - | - | (15) |
| P615L | Tail (KSP) | rs5763269 | Deleterious | Possibly Damaging | 0.357 | 15.7 | 55/175 | 27/100 | 0.196 | - | - | (2) |
|  |  |  |  |  |  |  | 84/188 | - | 0.2131 | - | - | (6) |
|  |  |  |  |  |  |  | 414/1126 | 205/613 |  | - | - | (11) |
|  |  |  |  |  |  |  | 30/371 | 66/711 | 0.07552 | - | OR 0.84 (95% CI 0.61-1.16); p=0.30 | (19) |
| T642M | Tail (KSP) | rs117258406 | Tolerated | Benign | 0.362 | 0.00100 | 1/46 | 18/699 | 0.008416 | SPG11 L1982S | OR 0.78 (95% CI 0.02-5.19); p=1.00 | (10) |
|  |  |  |  |  |  |  | 4/311 | 0/200 |  | - | - | (15) |
|  |  |  |  |  |  |  | 4/268 | - |  | - | - | (18) |
|  |  |  |  |  |  |  | 4/371 | 5/711 |  | - | - | (19) |
| K647N | Tail (KSP) | rs200634512 | Deleterious | Possibly Damaging | 0.365 | 14.7 | 1/5 | - | 0.000358 | - | - | (20) |
| V670E | Tail (KSP) | rs190692435 | Tolerated | Possibly Damaging | 0.170 | 0.240 | 2/371 | 5/711 | 0.02705 | - | OR 0.77 (95% CI 0.15-3.96); p=1.00 | (19) |
| A672E | Tail (KSP) | rs775497149 | Tolerated | Benign | 0.178 | 1.65 | 1/371 | 5/711 | 0.02376 | - | OR 0.38 (95% CI 0.05-3.28); p=0.67 | (19) |
| V726L | Tail (KSP) | rs1051285707 | Tolerated | Benign | 0.238 | 13.5 | 1/120 | - | 0.00003891 | - | - | (13) |
| P777L | Tail (KSP) | rs199748453 | Deleterious | Probably Damaging | 0.476 | 24.1 | 1/100 | - | 0.00005831 | ALS2 T293I | - | (21) |
| S787R | Tail (KSP) | rs568759161 | Deleterious | Probably Damaging | 0.211 | 16.06 | 2/268 | - | 0.002405 | - | - | (18) |
|  |  |  |  |  |  |  | 9/671 | 2/1787 |  | - | Discovery: OR 9.64 (95% CI 1.12-82.67); p=0.02 Replication: OR 14.43 (95% CI 1.61-129.40); p=0.009 Combined: OR 12.06 (95% CI 2.60-55.88); p=0.0003 | (19) |
| A805E | Tail (KSP) | - | - | - | - | - | 80/100 | - | N/A | - | - | (2) |
| E805A | Tail (KSP) | rs165602 | Deleterious | Possibly Damaging | 0.382 | 25.5 | 45/176 | - | 0.1551 | - | - | (6) |
|  |  |  |  |  |  |  | 309/1126 | 152/613 | 0.04673 | - | - | (11) |
|  |  |  |  |  |  |  | 10/371 | 30/711 |  | - | - | (19) |
| P848S | Tail (KEP) | - | Deleterious | Benign | 0.295 | 21.2 | 2/268 | - | N/A | - | - | (18) |
| K857R | Tail (KEP) | rs747689406 | Deleterious | Possibly Damaging | 0.325 | 23.0 | 1/311 | 0/200 | 0.00007522 | - | - | (15) |
| K867N | Tail (KEP) | rs138156220 | Deleterious | Probably Damaging | 0.427 | 21.2 | 1/182 | 0/190 | 0 | - | - | (6) |
|  |  |  |  |  |  |  | 1/5 | - | 0 | SOD1 G93V | - | (20) |
| E868K | Tail (KEP) | rs1295979036 | Tolerated | Probably Damaging | 0.578 | 25.3 | 1/45 | - | 0 | - | - | (16) |
| T905I | Tail (KEP) | rs202199780 | Tolerated | Benign | 0.187 | 10.1 | 2/268 | - | 0.001673 | - | - | (18) |
| K917E | Tail (KEP) | - | Deleterious | Possibly Damaging | 0.365 | 23.7 | 1/311 | 0/200 | 0 | CHMP2B A138T | - | (15) |
| E918G | Tail (KEP) | rs189881592 | Deleterious | Possibly Damaging | 0.330 | 23.8 | 1/186 | 0/190 | 0 | - | - | (6) |
|  |  |  |  |  |  |  | 1/311 | 0/200 | 0.003143 | - | - | (15) |
|  |  |  |  |  |  |  | 2/268 | - |  | - | - | (18) |
|  |  |  |  |  |  |  | 2/371 | 2/711 |  | - | OR 1.92 (95% CI 0.27-13.65); p=0.61 | (19) |
| Q117Ter | Rod (Coil 1A) | rs1317368575 | - | - | - | 37.0 | 1/371 | 0/711 | 0.00009524 | - | OR Inf (95% CI Na-Inf); p=0.34 | (19) |
| L158L | Rod (Coil 1B) | rs1002065392 | - | - | - | - | 1/75 | 1/50 | 0 | - | - | (2) |
| L349L | Rod (Coil 2B) | rs1269206770 | - | - | - | - | 1/186 | - | N/A | - | - | (6) |
| A400A | Rod (Coil 2B) | rs165734 | - | - | - | - | 10/371 | 30/711 | 0.1807 | - | - | (19) |
| A401A | Rod (Coil 2B) | - | - | - | - | - | 2x lower than in controls | 2x higher than in ALS | N/A | - | - | (2) |
| S580S | Tail (KSP) | rs114263951 | - | - | - | - | 5/188 | - | 0.01183 | - | - | (6) |
| A744A | Tail (KSP) | rs165923 | - | - | - | - | 104/185 | - | 0.7855 | - | - | (6) |
| D919D | Tail (KEP) | rs56200920 | - | - | - | - | 1/186 | - | 0.005927 | - | - | (6) |
|  |  |  |  |  |  |  | 2/371 | 10/711 | 0.01124 | - | - | (19) |
| V928V | Tail (KEP) | rs165625 | - | - | - | - | 149/186 | - | 0.7856 | - | - | (6) |
|  |  |  |  |  |  |  | 30/371 | 67/711 | 0.9238 | - | - | (19) |

Supplementary Table 3. Variant characteristics of all included records identified from the systematic review. All variants supported by case-control studies (34) were subject to subgroup-level meta-analysis, with 8 going forward to variant-level meta-analysis as the mutation was supported by at least 2 case-control studies. N/A values represent instances where the variant was not present in the population-specific gnomAD non-neuro database.

| **Variant** | **Start** | **End** | **Ref** | **Alt** |
| --- | --- | --- | --- | --- |
| E459Gfs*7 | 29885004 | 29885004 | GAACA | G |
| A528_P561del | 29885211 | 29885211 | GCTGAGGCCAAGTCCCCAGAGAAGGAGGAAGCAAAATCCCCAGCCGAAGTCAAGTCCCCTGAGAAGGCC | G |
| A652_K657del | 29885576 | 29885576 | TGAGAAGGCCAAGTCCCC | T |
| E658_K665del | 29885580 | 29885580 | AAGGCCAAGTCCCCAGAGAAGGAAG | A |
| E664_P669del | 29885604 | 29885604 | GAGGCCAAGTCCCCTGAGA | G |
| A686_K699del | 29885609 | 29885609 | CAAGTCCCCTGAGAAGGCCAAGTCCCCAGTGAAGGCAGAAGCA | C |
| S752_K757del | 29885858 | 29885858 | AGCTAAGTCCCCAGAGAAG | A |
| K790del | 29885996 | 29885996 | CAAG | C |
| K857del | 29886192 | 29886192 | GAGA | G |
| S704_K731dup (A708ins) | 29885709 | 29885709 | GCCAAGTCCCCAGTGAAGGAAGAAGCAAAGTCCCCTGAGAAGGCCAAGTCCCCAGTGAAGGAAGAAGCAAAGTCCCCTGAGAAG | G |
| A40V | 29876370 | 29876370 | C | T |
| A90V | 29876520 | 29876520 | C | T |
| R148P | 29876694 | 29876694 | G | C |
| E152D | 29876707 | 29876707 | G | C |
| Q171H | 29876764 | 29876764 | G | C |
| D187N | 29876810 | 29876810 | G | A |
| R192P | 29876826 | 29876826 | G | C |
| A203P | 29876858 | 29876858 | G | C |
| G249S | 29876996 | 29876996 | G | A |
| S285R | 29877106 | 29877106 | C | A |
| A314V | 29879421 | 29879421 | C | T |
| T338I | 29879493 | 29879493 | C | T |
| R346H | 29879517 | 29879517 | G | A |
| R352S | 29879534 | 29879534 | C | A |
| A380T | 29881766 | 29881766 | G | A |
| A400V | 29881827 | 29881827 | C | T |
| R412Q | 29884864 | 29884864 | G | A |
| E460K | - | - | - | - |
| E463K | 29885016 | 29885016 | G | A |
| E491K | 29885100 | 29885100 | G | A |
| P505L | 29885143 | 29885143 | C | T |
| P512S | 29885163 | 29885163 | C | T |
| E597D | 29885420 | 29885420 | G | C |
| P615L | 29885473 | 29885473 | C | T |
| T642M | 29885554 | 29885554 | C | T |
| K647N | 29885570 | 29885570 | G | C |
| V670E | 29885638 | 29885638 | T | A |
| A672E | 29885644 | 29885644 | C | A |
| V726L | 29885805 | 29885805 | G | C |
| P777L | 29885959 | 29885959 | C | T |
| S787R | 29885990 | 29885990 | C | G |
| A805E | 29886043 | 29886043 | C | A |
| E805A | 29886043 | 29886043 | A | C |
| P848S | 29886171 | 29886171 | C | T |
| K857R | 29886199 | 29886199 | A | G |
| K867N | 29886230 | 29886230 | G | T |
| E868K | 29886231 | 29886231 | G | A |
| T905I | 29886343 | 29886343 | C | T |
| K917E | 29886378 | 29886378 | A | G |
| E918G | 29886382 | 29886382 | A | G |
| Q117Ter | 29876600 | 29876600 | C | T |
| L158L | 29876725 | 29876725 | G | A |
| L349L | 29879527 | 29879527 | G | T |
| A400A | 29881828 | 29881828 | C | T |
| A401A | - | - | - | - |
| S580S | 29885369 | 29885369 | C | T |
| A744A | 29885861 | 29885861 | T | C |
| D919D | 29886386 | 29886386 | C | T |
| V928V | 29886413 | 29886413 | A | G |

Supplementary Table 4. Genomic coordinates and base pair substitutions (hg19) of all variants identified from the systematic review.

| **Variant** | **No. Studies** | **N Cases** | **N Controls** | **Fixed OR**  **(95% CI; p-value)** | **Random OR**  **(95% CI; p-value)** | **Heterogeneity**  **(Q statistic; p-value)** | **Heterogeneity**  **(I-squared)** | **Publication Bias (Egger t statistic; p-value)** | **Publication Bias (Harbord t statistic; p-value)** |
| --- | --- | --- | --- | --- | --- | --- | --- | --- | --- |
| G249S | 2 | 471 | 811 | 0.91  (0.28-2.94; 0.87) | 0.89  (0.26-3.03; 0.85) | 0.66 (0.42) | 0% | N/A | N/A |
| A380T | 3 | 782 | 1011 | 1.12  (0.39-3.21; 0.83) | 1.06  (0.34-3.33; 0.92) | 1.26 (0.53) | 0% | 12.17; **0.05** | 5.01; 0.13 |
| P615L | 3 | 1672 | 1424 | 1.11  (0.93-1.33; 0.24) | 1.11  (0.93-1.33; 0.24) | 1.54 (0.46) | 0% | -0.46; 0.73 | -0.46; 0.73 |
| T642M | 3 | 728 | 1610 | 1.71  (0.66-4.42; 0.27) | 1.56  (0.55-4.39; 0.40) | 1.14 (0.57) | 0% | 0.56; 0.67 | 0.35; 0.79 |
| E658_K665del | 2 | 578 | 930 | 0.91  (0.24-3.45; 0.88) | 0.88  (0.21-3.70; 0.86) | 0.78 (0.38) | 0% | N/A | N/A |
| S752_K757del | 2 | 694 | 939 | 1.03  (0.73-1.47; 0.85) | 1.04  (0.73-1.47; 0.84) | 0.80 (0.37) | 0% | N/A | N/A |
| K790del | 2 | 1482 | 919 | 1.56  (0.37-6.64; 0.55) | 1.31  (0.05-35.02; 0.87) | 3.03 (0.08) | **67%** | N/A | N/A |
| E805A | 2 | 1497 | 1324 | 1.08  (0.88-1.34; 0.46) | 0.94  (0.54-1.64; 0.83) | 2.40 (0.12) | **58%** | N/A | N/A |
| E918G | 3 | 868 | 1101 | 2.16  (0.49-9.46; 0.31) | 2.13  (0.48-9.40; 0.32) | 0.06 (0.97) | 0% | 0.78; 0.58 | 4.18; 0.15 |

Supplementary Table 5. Results of the variant-level meta-analysis. N/A values represent instances where publication bias could not be calculated as the minimum number of studies used for calculation was not reached.

| **MAF Category** | **Subgroup** | **No. Studies** | **N Cases** | **N Controls** | **Fixed OR (95% CI; p-value)** | **Random OR (95% CI; p-value)** | **Heterogeneity (Q statistic; p-value)** | **Heterogeneity (I-squared)** | **Publication Bias (Egger t statistic; p-value)** | **Publication Bias (Harbord t statistic; p-value)** |
| --- | --- | --- | --- | --- | --- | --- | --- | --- | --- | --- |
| Ultra-Rare (<0.1%) | All | 10 | 2729 | 2483 | 2.64  (1.22-5.68; **0.01**) | 2.27  (0.99-5.18; 0.05) | 5.12 (0.82) | 0% | 2.17; 0.06 | 1.13; 0.29 |
|  | Tail | 7 | 1918 | 2063 | 2.01  (0.80-5.05; 0.14) | 1.82  (0.68-4.87; 0.24) | 3.06 (0.80) | 0% | 4.38; **7.2E-03** | 3.01; **0.03** |
|  | Missense | 6 | 1679 | 1521 | 4.35  (1.39-13.64; **0.01**) | 4.14  (1.30-13.18; **0.02**) | 1.17 (0.95) | 0% | -0.29; 0.78 | 0.40; 0.71 |
|  | Head Missense | 2 | 285 | 290 | 1.70  (0.22-12.95; 0.61) | 1.63  (0.20-13.35; 0.65) | 0.25 (0.62) | 0% | N/A | N/A |
|  | Rod Missense | 5 | 1368 | 1321 | 3.28  (0.82-13.15; 0.09) | 3.17  (0.78-12.90; 0.11) | 0.77 (0.94) | 0% | -0.92; 0.43 | -0.76; 0.50 |
|  | Tail Missense | 3 | 868 | 1101 | 5.05  (0.84-30.22; 0.08) | 5.10  (0.87-29.98; 0.07) | 0.01 (0.99) | 0% | 3.88; 0.16 | 15.36; **0.04** |
|  | Tail Deletion | 3 | 886 | 753 | 0.97  (0.28-3.40; 0.97) | 0.94  (0.26-3.38; 0.93) | 0.52 (0.77) | 0% | 2.25; 0.27 | 2.45; 0.25 |
| High-Frequency Rare (0.1-1%) | All | 7 | 2764 | 3106 | 2.11  (1.23-3.62; **6.5E-03**) | 2.16  (0.77-6.10; 0.15) | 9.92 (0.13) | 40% | 0.55; 0.61 | 0.45; 0.67 |
|  | Missense | 5 | 1282 | 2187 | 2.23  (1.25-3.97; **6.7E-03**) | 2.53  (0.82-7.85; 0.11) | 6.49 (0.17) | 38% | 0.80; 0.48 | 0.79; 0.49 |
|  | Tail | 5 | 2464 | 2906 | 3.14  (1.56-6.31; **1.3E-03**) | 3.26  (0.99-10.74; 0.05) | 6.64 (0.16) | 40% | 0.30; 0.79 | 0.17; 0.88 |
|  | Rod Missense | 4 | 982 | 1111 | 1.01  (0.42-2.44; 0.98) | 0.95  (0.38-2.39, 0.91) | 1.58 (0.66) | 0% | 1.30; 0.32 | 1.39; 0.30 |
|  | Tail Missense | 3 | 982 | 1987 | 3.91  (1.77-8.64; **7.0E-04**) | 4.44  (1.41-13.96; **0.01**) | 2.16 (0.34) | 7% | 1.81; 0.32 | 1.93; 0.30 |
| Rare (< 1%) | All | 13 | 4355 | 4272 | 2.54  (1.59-4.06; **< 1.0E-04**) | 2.28  (1.38-3.77; **1.3E-03**) | 12.08 (0.44) | 1% | 0.23; 0.82 | 0.12; 0.90 |
|  | Tail | 9 | 3344 | 3752 | 2.83  (1.59-5.04; **4.0E-04**) | 2.57  (1.10-5.98; **0.03**) | 10.80 (0.21) | 26% | 0.30; 0.78 | 0.03; 0.98 |
|  | Tail (exc. Missense) | 5 | 2176 | 1575 | 1.37  (0.54-3.43; 0.51) | 1.05  (0.37-3.01; 0.93) | 4.32 (0.36) | 7% | 1.22; 0.31 | 0.16; 0.88 |
|  | Missense | 8 | 2179 | 2697 | 2.78  (1.64-4.71; **< 1.0E-04**) | 2.60  (1.34-5.10; **4.8E-03**) | 7.52 (0.38) | 7% | 0.93; 0.39 | 0.89; 0.41 |
|  | Missense (exc. Tail) | 7 | 1879 | 1621 | 1.57  (0.77-3.21; 0.22) | 1.44  (0.68-3.05; 0.34) | 2.71 (0.84) | 0% | 1.22; 0.28 | 1.44; 0.21 |
|  | Rod Missense | 7 | 1879 | 1621 | 1.51  (0.72-3.15; 0.28) | 1.41  (0.67-3.04; 0.38) | 2.37 (0.89) | 0% | 1.33; 0.24 | 1.97; 0.11 |
|  | Tail Missense | 4 | 1168 | 2177 | 4.55  (2.13-9.71; **< 1.0E-04**) | 4.65  (1.79-12.11; **1.6E-03**) | 2.30 (0.51) | 0% | 2.07; 0.17 | 1.82; 0.21 |
|  | Tail Deletion | 4 | 2012 | 1366 | 1.22  (0.46-3.22; 0.68) | 0.90  (0.30-2.74; 0.85) | 3.62 (0.31) | 17% | 0.75; 0.53 | -0.50; 0.66 |
| Common (> 1%) | All | 6 | 2025 | 2442 | 1.11  (0.94-1.30; 0.21) | 1.20  (0.87-1.65; 0.26) | 9.30 (0.10) | 46% | 0.99; 0.38 | 1.13; 0.32 |
|  | Tail | 6 | 2025 | 2442 | 1.11  (0.95-1.31; 0.19) | 1.20  (0.88-1.63; 0.25) | 8.95 (0.11) | 44% | 1.00; 0.37 | 1.14; 0.32 |
|  | Missense | 5 | 1818 | 2223 | 1.12  (0.94-1.34; 0.20) | 1.18  (0.79-1.78; 0.42) | 10.12 (**0.04**) | **61%** | 0.32; 0.77 | 0.34; 0.76 |
|  | Tail Missense | 5 | 1818 | 2223 | 1.13  (0.95-1.36; 0.17) | 1.19  (0.79-1.77; 0.40) | 9.62 (**0.05**) | **58%** | 0.30; 0.78 | 0.32; 0.77 |
|  | Tail Deletion | 2 | 578 | 930 | 1.03  (0.78-1.35; 0.85) | 1.03  (0.78-1.35; 0.86) | 0.48 (0.49) | 0% | N/A | N/A |
| All | Rod Missense | 7 | 1879 | 1721 | 1.26  (0.67-2.37; 0.48) | 1.18  (0.61-2.29; 0.62) | 2.69 (0.85) | 0% | 1.82; 0.13 | 2.42; 0.06 |
|  | Tail Missense | 8 | 2615 | 3689 | 1.24  (1.04-1.48; **0.02**) | 1.45  (0.96-2.19; 0.08) | 14.58 (**0.04**) | **52%** | 2.20; 0.07 | 2.41; 0.05 |
|  | Tail Deletion | 5 | 2383 | 2077 | 1.04  (0.80-1.35; 0.77) | 1.02  (0.78-1.33; 0.89) | 4.02 (0.40) | 1% | 0.29; 0.79 | 0.35; 0.75 |

Supplementary Table 6. Results of the subgroup meta-analysis of variants identified from the systematic review that were supported by two or more case-control studies. N/A values represent instances where publication bias could not be calculated as the minimum number of studies required for calculation was not reached.

| **Start on chr 22** | **Ref** | **Alt** | **Variant Type** | **VEP Impact** | **Exon** | **Intron** | **HGVS.c** | **HGVS.p** | **rsID** |
| --- | --- | --- | --- | --- | --- | --- | --- | --- | --- |
| 29876227 | C | G | 5' UTR | Modifier | 1 | - | c.-25C>G | - | - |
| 29876229 | G | A | 5' UTR | Modifier | 1 | - | c.-23G>A | - | rs1280164847 |
| 29876235 | G | C | 5' UTR | Modifier | 1 | - | c.-17G>C | - | - |
| 29876236 | C | A | 5' UTR | Modifier | 1 | - | c.-16C>A | - | rs996402317 |
| 29876272 | G | A | Synonymous | Low | 1 | - | c.21G>A | p.Ala7Ala | rs192350983 |
| 29876298 | C | A | Missense | Moderate | 1 | - | c.47C>A | p.Ala16Glu | rs1468605569 |
| 29876336 | G | T | Missense | Moderate | 1 | - | c.85G>T | p.Ala29Ser | rs752311867 |
| 29876338 | C | T | Synonymous | Low | 1 | - | c.87C>T | p.Ala29Ala | - |
| 29876348 | G | A | Missense | Moderate | 1 | - | c.97G>A | p.Gly33Ser | - |
| 29876392 | C | T | Synonymous | Low | 1 | - | c.141C>T | p.Phe47Phe | rs780966618 |
| 29876395 | C | G | Missense | Moderate | 1 | - | c.144C>G | p.His48Gln | rs1325065979 |
| 29876404 | A | G | Synonymous | Low | 1 | - | c.153A>G | p.Thr51Thr | rs755886692 |
| 29876410 | G | A | Synonymous | Low | 1 | - | c.159G>A | p.Thr53Thr | rs1205368020 |
| 29876416 | G | A | Synonymous | Low | 1 | - | c.165G>A | p.Val55Val | rs897546803 |
| 29876473 | C | T | Synonymous | Low | 1 | - | c.222C>T | p.Thr74Thr | rs1413495861 |
| 29876520 | C | T | Missense | Moderate | 1 | - | c.269C>T | p.Ala90Val | rs61556467 |
| 29876529 | C | T | Missense | Moderate | 1 | - | c.278C>T | p.Thr93Ile | rs751160327 |
| 29876552 | C | T | Synonymous | Low | 1 | - | c.301C>T | p.Leu101Leu | rs1231899865 |
| 29876555 | C | T | Stop Gained | High | 1 | - | c.304C>T | p.Gln102Ter | rs948554818 |
| 29876609 | G | A | Missense | Moderate | 1 | - | c.358G>A | p.Ala120Thr | rs1386911340 |
| 29876620 | C | T | Synonymous | Low | 1 | - | c.369C>T | p.Arg123Arg | rs768460278 |
| 29876632 | C | G | Synonymous | Low | 1 | - | c.381C>G | p.Gly127Gly | rs760591823 |
| 29876649 | G | A | Missense | Moderate | 1 | - | c.398G>A | p.Arg133Gln | rs997535105 |
| 29876660 | G | T | Missense | Moderate | 1 | - | c.409G>T | p.Ala137Ser | rs754799725 |
| 29876668 | C | G | Synonymous | Low | 1 | - | c.417C>G | p.Arg139Arg | rs912176188 |
| 29876699 | G | A | Missense | Moderate | 1 | - | c.448G>A | p.Val150Ile | rs769162779 |
| 29876744 | G | A | Missense | Moderate | 1 | - | c.493G>A | p.Gly165Ser | - |
| 29876747 | C | T | Stop Gained | High | 1 | - | c.496C>T | p.Gln166Ter | - |
| 29876754 | G | T | Missense | Moderate | 1 | - | c.503G>T | p.Arg168Leu | rs1009899708 |
| 29876756 | C | T | Synonymous | Low | 1 | - | c.505C>T | p.Leu169Leu | rs1277092292 |
| 29876761 | G | A | Synonymous | Low | 1 | - | c.510G>A | p.Glu170Glu | rs1208767813 |
| 29876765 | G | A | Missense | Moderate | 1 | - | c.514G>A | p.Glu172Lys | rs1176398662 |
| 29876777 | G | A | Missense | Moderate | 1 | - | c.526G>A | p.Glu176Lys | rs1422942222 |
| 29876800 | G | A | Synonymous | Low | 1 | - | c.549G>A | p.Gln183Gln | - |
| 29876834 | G | A | Missense | Moderate | 1 | - | c.583G>A | p.Ala195Thr | rs1273011909 |
| 29876842 | G | C | Synonymous | Low | 1 | - | c.591G>C | p.Ala197Ala | rs1459116357 |
| 29876845 | G | A | Synonymous | Low | 1 | - | c.594G>A | p.Ala198Ala | rs748478910 |
| 29876857 | CGCGCGCTG | - | Frameshift Deletion | High | 1 | - | c.606_613del | p.Ala203ArgfsTer74 | - |
| 29876866 | C | T | Synonymous | Low | 1 | - | c.615C>T | p.Phe205Phe | rs1301849129 |
| 29876878 | C | G | Synonymous | Low | 1 | - | c.627C>G | p.Ala209Ala | rs562497163 |
| 29876927 | G | A | Missense | Moderate | 1 | - | c.676G>A | p.Glu226Lys | - |
| 29876952 | A | C | Missense | Moderate | 1 | - | c.701A>C | p.His234Pro | - |
| 29876955 | A | T | Missense | Moderate | 1 | - | c.704A>T | p.Gln235Leu | - |
| 29876977 | C | G | Synonymous | Low | 1 | - | c.726C>G | p.Leu242Leu | rs560344725 |
| 29876978 | G | A | Missense | Moderate | 1 | - | c.727G>A | p.Gly243Ser | rs548521745 |
| 29876983 | G | A | Synonymous | Low | 1 | - | c.732G>A | p.Gln244Gln | rs527475764 |
| 29876996 | G | A | Missense | Moderate | 1 | - | c.745G>A | p.Gly249Ser | rs60825978 |
| 29877028 | G | T | Synonymous | Low | 1 | - | c.777G>T | p.Thr259Thr | rs915610438 |
| 29877033 | A | G | Missense | Moderate | 1 | - | c.782A>G | p.Asp261Gly | rs768531339 |
| 29877061 | G | C | Synonymous | Low | 1 | - | c.810G>C | p.Ala270Ala | rs201868609 |
| 29877094 | C | G | Missense | Moderate | 1 | - | c.843C>G | p.His281Gln | rs1267522365 |
| 29877099 | T | C | Missense | Moderate | 1 | - | c.848T>C | p.Val283Ala | - |
| 29877102 | A | G | Missense | Moderate | 1 | - | c.851A>G | p.Gln284Arg | rs746595090 |
| 29877107 | A | G | Missense | Moderate | 1 | - | c.856A>G | p.Thr286Ala | rs770443491 |
| 29877109 | G | C | Synonymous | Low | 1 | - | c.858G>C | p.Thr286Thr | rs1477253596 |
| 29877142 | G | A | Intronic | Low | - | 1 | c.883+8G>A | - | rs1191279791 |
| 29877150 | G | A | Intronic | Modifier | - | 1 | c.883+16G>A | - | rs534281794 |
| 29877158 | G | C | Intronic | Modifier | - | 1 | c.883+24G>C | - | rs1359878602 |
| 29877205 | C | G | Intronic | Modifier | - | 1 | c.883+71C>G | - | rs1474258062 |
| 29877208 | A | G | Intronic | Modifier | - | 1 | c.883+74A>G | - | - |
| 29877222 | G | A | Intronic | Modifier | - | 1 | c.883+88G>A | - | rs567088364 |
| 29877234 | A | G | Intronic | Modifier | - | 1 | c.883+100A>G | - | - |
| 29877239 | C | T | Intronic | Modifier | - | 1 | c.883+105C>T | - | rs1414146401 |
| 29877256 | C | T | Intronic | Modifier | - | 1 | c.883+122C>T | - | rs534144890 |
| 29877263 | A | G | Intronic | Modifier | - | 1 | c.883+129A>G | - | rs138081293 |
| 29877317 | C | T | Intronic | Modifier | - | 1 | c.883+183C>T | - | - |
| 29877321 | A | G | Intronic | Modifier | - | 1 | c.883+187A>G | - | rs367951665 |
| 29877324 | C | T | Intronic | Modifier | - | 1 | c.883+190C>T | - | - |
| 29877343 | C | T | Intronic | Modifier | - | 1 | c.883+209C>T | - | rs1373636600 |
| 29877344 | C | T | Intronic | Modifier | - | 1 | c.883+210C>T | - | rs573999650 |
| 29877346 | C | G | Intronic | Modifier | - | 1 | c.883+212C>G | - | - |
| 29877367 | G | A | Intronic | Modifier | - | 1 | c.883+233G>A | - | rs56083406 |
| 29877370 | C | T | Intronic | Modifier | - | 1 | c.883+236C>T | - | - |
| 29877386 | C | T | Intronic | Modifier | - | 1 | c.883+252C>T | - | - |
| 29877417 | C | T | Intronic | Modifier | - | 1 | c.883+283C>T | - | rs165607 |
| 29877432 | C | T | Intronic | Modifier | - | 1 | c.883+298C>T | - | rs556602600 |
| 29877437 | C | CGTCAAGAAAAAAACAAGTTT | Intronic | Modifier | - | 1 | c.883+305_883+325dup | - | rs760534140 |
| 29877441 | C | G | Intronic | Modifier | - | 1 | c.883+307C>G | - | rs73407035 |
| 29877444 | G | A | Intronic | Modifier | - | 1 | c.883+317dup | - | rs763994664 |
| 29877448 | A | G | Intronic | Modifier | - | 1 | c.883+314A>G | - | - |
| 29877475 | G | A | Intronic | Modifier | - | 1 | c.883+341G>A | - | rs576631246 |
| 29877541 | G | C | Intronic | Modifier | - | 1 | c.883+407G>C | - | - |
| 29877545 | A | G | Intronic | Modifier | - | 1 | c.883+411A>G | - | rs75956622 |
| 29877557 | C | T | Intronic | Modifier | - | 1 | c.883+423C>T | - | rs165697 |
| 29877565 | A | G | Intronic | Modifier | - | 1 | c.883+431A>G | - | rs165865 |
| 29877581 | T | A | Intronic | Modifier | - | 1 | c.883+447T>A | - | rs188853358 |
| 29877587 | C | T | Intronic | Modifier | - | 1 | c.883+453C>T | - | rs144739005 |
| 29877592 | C | A | Intronic | Modifier | - | 1 | c.883+458C>A | - | rs1218743924 |
| 29877620 | T | A | Intronic | Modifier | - | 1 | c.883+486T>A | - | - |
| 29877624 | C | T | Intronic | Modifier | - | 1 | c.883+490C>T | - | rs79658766 |
| 29877625 | A | G | Intronic | Modifier | - | 1 | c.883+491A>G | - | rs165821 |
| 29877656 | AC | - | Intronic | Modifier | - | 1 | c.883+522del | - | rs1168824251 |
| 29877657 | G | C | Intronic | Modifier | - | 1 | c.883+523G>C | - | rs78580989 |
| 29877693 | C | A | Intronic | Modifier | - | 1 | c.883+559C>A | - | - |
| 29877699 | G | A | Intronic | Modifier | - | 1 | c.883+565G>A | - | rs780172669 |
| 29877700 | G | A | Intronic | Modifier | - | 1 | c.883+566G>A | - | - |
| 29877710 | C | T | Intronic | Modifier | - | 1 | c.883+576C>T | - | - |
| 29877715 | G | A | Intronic | Modifier | - | 1 | c.883+581G>A | - | - |
| 29877716 | T | G | Intronic | Modifier | - | 1 | c.883+582T>G | - | - |
| 29877803 | A | G | Intronic | Modifier | - | 1 | c.883+669A>G | - | - |
| 29877809 | G | A | Intronic | Modifier | - | 1 | c.883+675G>A | - | rs1187445478 |
| 29877855 | G | T | Intronic | Modifier | - | 1 | c.883+721G>T | - | - |
| 29877860 | G | T | Intronic | Modifier | - | 1 | c.883+726G>T | - | rs73881537 |
| 29877874 | C | G | Intronic | Modifier | - | 1 | c.883+740C>G | - | - |
| 29877877 | C | TGCA | Intronic | Modifier | - | 1 | c.883+744_883+747dup | - | rs1265330728 |
| 29877882 | G | A | Intronic | Modifier | - | 1 | c.883+748G>A | - | rs561113494 |
| 29877893 | A | G | Intronic | Modifier | - | 1 | c.883+759A>G | - | - |
| 29877907 | T | G | Intronic | Modifier | - | 1 | c.883+773T>G | - | - |
| 29877961 | G | C | Intronic | Modifier | - | 1 | c.883+827G>C | - | - |
| 29877979 | C | T | Intronic | Modifier | - | 1 | c.883+845C>T | - | - |
| 29877999 | ACTGT | - | Intronic | Modifier | - | 1 | c.883+866_883+869del | - | rs1359000475 |
| 29878010 | CTT | - | Intronic | Modifier | - | 1 | c.883+876_883+877del | - | rs1341072419 |
| 29878010 | T | C | Intronic | Modifier | - | 1 | c.883+876T>C | - | rs563803182 |
| 29878026 | TGAGA | - | Intronic | Modifier | - | 1 | c.883+894_883+897del | - | rs918545488 |
| 29878039 | G | T | Intronic | Modifier | - | 1 | c.883+905G>T | - | rs747959620 |
| 29878057 | A | G | Intronic | Modifier | - | 1 | c.883+923A>G | - | - |
| 29878068 | A | T | Intronic | Modifier | - | 1 | c.883+934A>T | - | - |
| 29878168 | C | T | Intronic | Modifier | - | 1 | c.883+1034C>T | - | rs577782932 |
| 29878198 | T | C | Intronic | Modifier | - | 1 | c.883+1068dup | - | rs543584318 |
| 29878200 | C | T | Intronic | Modifier | - | 1 | c.883+1066C>T | - | rs1018018710 |
| 29878201 | C | T | Intronic | Modifier | - | 1 | c.883+1067C>T | - | rs115007285 |
| 29878318 | G | A | Intronic | Modifier | - | 1 | c.884-1046G>A | - | rs137916245 |
| 29878328 | G | A | Intronic | Modifier | - | 1 | c.884-1036G>A | - | rs776842833 |
| 29878367 | C | G | Intronic | Modifier | - | 1 | c.884-997C>G | - | rs980795548 |
| 29878435 | AGGTTTCCTGGGAACCACGAGCG | - | Intronic | Modifier | - | 1 | c.884-946_884-925del | - | - |
| 29878445 | C | G | Intronic | Modifier | - | 1 | c.884-914dup | - | rs148457788 |
| 29878448 | G | A | Intronic | Modifier | - | 1 | c.884-916_884-915insA | - | rs59399758 |
| 29878450 | G | C | Intronic | Modifier | - | 1 | c.884-914G>C | - | - |
| 29878452 | C | G | Intronic | Modifier | - | 1 | c.884-912C>G | - | rs759563670 |
| 29878481 | A | G | Intronic | Modifier | - | 1 | c.884-883A>G | - | rs114042882 |
| 29878483 | G | A | Intronic | Modifier | - | 1 | c.884-881G>A | - | rs995319069 |
| 29878525 | C | T | Intronic | Modifier | - | 1 | c.884-839C>T | - | rs982635274 |
| 29878581 | G | A | Intronic | Modifier | - | 1 | c.884-783G>A | - | - |
| 29878583 | C | T | Intronic | Modifier | - | 1 | c.884-781C>T | - | - |
| 29878629 | G | A | Intronic | Modifier | - | 1 | c.884-735G>A | - | rs990302434 |
| 29878670 | T | C | Intronic | Modifier | - | 1 | c.884-694T>C | - | rs114681751 |
| 29878698 | A | G | Intronic | Modifier | - | 1 | c.884-666A>G | - | - |
| 29878709 | C | T | Intronic | Modifier | - | 1 | c.884-655C>T | - | rs1050867473 |
| 29878820 | T | C | Intronic | Modifier | - | 1 | c.884-544T>C | - | - |
| 29878865 | G | A | Intronic | Modifier | - | 1 | c.884-499G>A | - | rs60499988 |
| 29878869 | C | T | Intronic | Modifier | - | 1 | c.884-495C>T | - | rs1268750543 |
| 29878874 | G | A | Intronic | Modifier | - | 1 | c.884-490G>A | - | - |
| 29878890 | C | T | Intronic | Modifier | - | 1 | c.884-474C>T | - | - |
| 29878894 | A | C | Intronic | Modifier | - | 1 | c.884-470A>C | - | - |
| 29878930 | G | T | Intronic | Modifier | - | 1 | c.884-434G>T | - | rs1046802224 |
| 29878947 | T | C | Intronic | Modifier | - | 1 | c.884-417T>C | - | rs957592448 |
| 29878961 | CAT | - | Intronic | Modifier | - | 1 | c.884-404_884-403del | - | rs1482065911 |
| 29878995 | C | T | Intronic | Modifier | - | 1 | c.884-369C>T | - | rs762182598 |
| 29879010 | C | T | Intronic | Modifier | - | 1 | c.884-354C>T | - | rs1012822803 |
| 29879022 | C | A | Intronic | Modifier | - | 1 | c.884-342C>A | - | rs143372377 |
| 29879036 | G | A | Intronic | Modifier | - | 1 | c.884-328G>A | - | rs942354319 |
| 29879044 | C | T | Intronic | Modifier | - | 1 | c.884-320C>T | - | rs1039764937 |
| 29879072 | G | A | Intronic | Modifier | - | 1 | c.884-292G>A | - | rs577074185 |
| 29879079 | G | A | Intronic | Modifier | - | 1 | c.884-285G>A | - | rs949238939 |
| 29879211 | C | T | Intronic | Modifier | - | 1 | c.884-153C>T | - | rs1249661164 |
| 29879212 | G | A | Intronic | Modifier | - | 1 | c.884-152G>A | - | rs1456261265 |
| 29879229 | C | T | Intronic | Modifier | - | 1 | c.884-135C>T | - | rs562037652 |
| 29879246 | C | T | Intronic | Modifier | - | 1 | c.884-118C>T | - | - |
| 29879247 | G | A | Intronic | Modifier | - | 1 | c.884-117G>A | - | rs112005621 |
| 29879251 | G | A | Intronic | Modifier | - | 1 | c.884-113G>A | - | rs1176098439 |
| 29879252 | C | T | Intronic | Modifier | - | 1 | c.884-112C>T | - | rs1359134672 |
| 29879264 | CT | - | Intronic | Modifier | - | 1 | c.884-100del | - | - |
| 29879264 | T | A | Intronic | Modifier | - | 1 | c.884-100_884-99insA | - | rs1291058402 |
| 29879281 | AAG | - | Intronic | Modifier | - | 1 | c.884-83_884-82del | - | rs1295712746 |
| 29879281 | AG | - | Intronic | Modifier | - | 1 | c.884-83del | - | - |
| 29879308 | C | T | Intronic | Modifier | - | 1 | c.884-56C>T | - | rs1019810165 |
| 29879343 | C | T | Intronic | Modifier | - | 1 | c.884-21C>T | - | rs1482031249 |
| 29879383 | G | A | Synonymous | Low | 2 | - | c.903G>A | p.Ser301Ser | rs781291855 |
| 29879402 | A | CAGACGCTATGCGCT | In-frame Insertion | Moderate | 2 | - | c.926_940dup | p.Asp309_Ser313dup | rs1380569251 |
| 29879421 | C | T | Missense | Moderate | 2 | - | c.941C>T | p.Ala314Val | rs539511579 |
| 29879444 | C | T | Missense | Moderate | 2 | - | c.964C>T | p.Arg322Trp | rs778265423 |
| 29879483 | C | G | Missense | Moderate | 2 | - | c.1003C>G | p.Leu335Val | - |
| 29879516 | C | T | Missense | Moderate | 2 | - | c.1036C>T | p.Arg346Cys | rs139219355 |
| 29879518 | C | G | Synonymous | Low | 2 | - | c.1038C>G | p.Arg346Arg | rs377262654 |
| 29879524 | G | C | Missense | Moderate | 2 | - | c.1044G>C | p.Glu348Asp | rs1475562664 |
| 29879535 | G | A | Missense | Moderate | 2 | - | c.1055G>A | p.Arg352His | rs750088241 |
| 29879545 | C | T | Synonymous | Low | 2 | - | c.1065C>T | p.Ala355Ala | rs144040465 |
| 29879559 | A | G | Missense | Moderate | 2 | - | c.1079A>G | p.Tyr360Cys | rs768679590 |
| 29879570 | CA | - | Intronic | Low | - | 2 | c.1083+7del | - | - |
| 29879592 | C | G | Intronic | Modifier | - | 2 | c.1083+29C>G | - | rs1357430644 |
| 29879601 | C | T | Intronic | Modifier | - | 2 | c.1083+38C>T | - | rs1020522395 |
| 29879604 | A | G | Intronic | Modifier | - | 2 | c.1083+41A>G | - | rs371191443 |
| 29879646 | G | T | Intronic | Modifier | - | 2 | c.1083+83G>T | - | rs570932481 |
| 29879653 | T | C | Intronic | Modifier | - | 2 | c.1083+90T>C | - | - |
| 29879659 | A | C | Intronic | Modifier | - | 2 | c.1083+96A>C | - | rs558830358 |
| 29879661 | A | T | Intronic | Modifier | - | 2 | c.1083+98A>T | - | rs775797988 |
| 29879662 | T | C | Intronic | Modifier | - | 2 | c.1083+99T>C | - | rs1004871512 |
| 29879671 | T | C | Intronic | Modifier | - | 2 | c.1083+108T>C | - | rs747239243 |
| 29879693 | CT | - | Intronic | Modifier | - | 2 | c.1083+132del | - | - |
| 29879695 | T | C | Intronic | Modifier | - | 2 | c.1083+132T>C | - | rs1403096338 |
| 29879745 | C | G | Intronic | Modifier | - | 2 | c.1083+182C>G | - | rs77685295 |
| 29879753 | C | T | Intronic | Modifier | - | 2 | c.1083+190C>T | - | - |
| 29879754 | C | T | Intronic | Modifier | - | 2 | c.1083+191C>T | - | rs114811985 |
| 29879817 | GATAGACTT | - | Intronic | Modifier | - | 2 | c.1083+247_1083+254del | - | - |
| 29879828 | A | TTTATTTT | Intronic | Modifier | - | 2 | c.1083+272_1083+273insTTTTATTT | - | - |
| 29879841 | TTTATTTAT | A | Intronic | Modifier | - | 2 | c.1083+270_1083+278delinsA | - | - |
| 29879844 | A | TTTTTTTT | Intronic | Modifier | - | 2 | c.1083+284_1083+285insTTTTTTTT | - | - |
| 29879847 | T | TGAG | Intronic | Modifier | - | 2 | c.1083+284_1083+285insTGAG | - | - |
| 29879853 | T | TTATTTAC | Intronic | Modifier | - | 2 | c.1083+292_1083+293insATTTACTT | - | rs1342137926 |
| 29879888 | G | T | Intronic | Modifier | - | 2 | c.1083+325G>T | - | - |
| 29879914 | C | T | Intronic | Modifier | - | 2 | c.1083+351C>T | - | - |
| 29879917 | C | A | Intronic | Modifier | - | 2 | c.1083+354C>A | - | rs34915139 |
| 29879923 | C | T | Intronic | Modifier | - | 2 | c.1083+360C>T | - | - |
| 29879943 | A | G | Intronic | Modifier | - | 2 | c.1083+380A>G | - | rs969772763 |
| 29879946 | TTC | - | Intronic | Modifier | - | 2 | c.1083+384_1083+385del | - | - |
| 29879948 | C | T | Intronic | Modifier | - | 2 | c.1083+385C>T | - | rs761560241 |
| 29879963 | C | T | Intronic | Modifier | - | 2 | c.1083+400C>T | - | rs903216611 |
| 29879970 | C | T | Intronic | Modifier | - | 2 | c.1083+407C>T | - | rs369540636 |
| 29879979 | C | T | Intronic | Modifier | - | 2 | c.1083+416C>T | - | rs899973194 |
| 29879984 | G | A | Intronic | Modifier | - | 2 | c.1083+421G>A | - | rs988563535 |
| 29880013 | A | G | Intronic | Modifier | - | 2 | c.1083+450A>G | - | rs545472872 |
| 29880049 | G | A | Intronic | Modifier | - | 2 | c.1083+486G>A | - | rs8136014 |
| 29880091 | A | G | Intronic | Modifier | - | 2 | c.1083+528A>G | - | rs1003956839 |
| 29880114 | C | T | Intronic | Modifier | - | 2 | c.1083+551C>T | - | - |
| 29880119 | G | T | Intronic | Modifier | - | 2 | c.1083+556G>T | - | - |
| 29880128 | C | T | Intronic | Modifier | - | 2 | c.1083+565C>T | - | rs148546902 |
| 29880129 | G | A | Intronic | Modifier | - | 2 | c.1083+566G>A | - | rs868328509 |
| 29880141 | A | AGAGAT | Intronic | Modifier | - | 2 | c.1083+580_1083+585dup | - | rs1321513053 |
| 29880155 | TA | - | Intronic | Modifier | - | 2 | c.1083+594del | - | - |
| 29880167 | T | C | Intronic | Modifier | - | 2 | c.1083+604T>C | - | rs778934270 |
| 29880187 | C | T | Intronic | Modifier | - | 2 | c.1083+624C>T | - | rs959441263 |
| 29880193 | G | T | Intronic | Modifier | - | 2 | c.1083+630G>T | - | - |
| 29880194 | G | A | Intronic | Modifier | - | 2 | c.1083+631G>A | - | rs117259769 |
| 29880211 | C | T | Intronic | Modifier | - | 2 | c.1083+648C>T | - | rs77580710 |
| 29880233 | T | C | Intronic | Modifier | - | 2 | c.1083+670T>C | - | rs1458642140 |
| 29880260 | C | T | Intronic | Modifier | - | 2 | c.1083+697C>T | - | rs537568541 |
| 29880261 | G | A | Intronic | Modifier | - | 2 | c.1083+698G>A | - | rs57986874 |
| 29880277 | A | G | Intronic | Modifier | - | 2 | c.1083+714A>G | - | rs189299986 |
| 29880346 | C | T | Intronic | Modifier | - | 2 | c.1083+783C>T | - | rs552866413 |
| 29880382 | A | C | Intronic | Modifier | - | 2 | c.1083+819A>C | - | - |
| 29880386 | T | C | Intronic | Modifier | - | 2 | c.1083+823T>C | - | rs556568863 |
| 29880400 | G | A | Intronic | Modifier | - | 2 | c.1083+837G>A | - | rs1451269879 |
| 29880445 | A | G | Intronic | Modifier | - | 2 | c.1083+882A>G | - | rs572804768 |
| 29880458 | G | T | Intronic | Modifier | - | 2 | c.1083+895G>T | - | rs924730895 |
| 29880492 | A | G | Intronic | Modifier | - | 2 | c.1083+929A>G | - | rs151051572 |
| 29880502 | G | A | Intronic | Modifier | - | 2 | c.1083+939G>A | - | rs2240634 |
| 29880528 | G | A | Intronic | Modifier | - | 2 | c.1083+965G>A | - | rs563717409 |
| 29880580 | G | C | Intronic | Modifier | - | 2 | c.1083+1017G>C | - | - |
| 29880585 | G | A | Intronic | Modifier | - | 2 | c.1083+1022G>A | - | rs543290585 |
| 29880591 | G | A | Intronic | Modifier | - | 2 | c.1083+1028G>A | - | rs1366926907 |
| 29880621 | C | T | Intronic | Modifier | - | 2 | c.1083+1058C>T | - | rs534776514 |
| 29880630 | G | A | Intronic | Modifier | - | 2 | c.1083+1067G>A | - | rs1019218040 |
| 29880652 | T | A | Intronic | Modifier | - | 2 | c.1084-1053dup | - | rs773168247 |
| 29880653 | A | T | Intronic | Modifier | - | 2 | c.1084-1059A>T | - | rs965488701 |
| 29880662 | A | T | Intronic | Modifier | - | 2 | c.1084-1050A>T | - | rs180872873 |
| 29880665 | TAAAA | - | Intronic | Modifier | - | 2 | c.1084-1047_1084-1044del | - | rs1273203992 |
| 29880692 | C | T | Intronic | Modifier | - | 2 | c.1084-1020C>T | - | - |
| 29880693 | C | G | Intronic | Modifier | - | 2 | c.1084-1019C>G | - | rs567894563 |
| 29880694 | T | C | Intronic | Modifier | - | 2 | c.1084-1018T>C | - | rs984314659 |
| 29880705 | C | G | Intronic | Modifier | - | 2 | c.1084-1007C>G | - | rs760470586 |
| 29880759 | G | A | Intronic | Modifier | - | 2 | c.1084-953G>A | - | - |
| 29880765 | T | C | Intronic | Modifier | - | 2 | c.1084-947T>C | - | rs939108695 |
| 29880766 | CTG | - | Intronic | Modifier | - | 2 | c.1084-945_1084-944del | - | rs770692971 |
| 29880766 | G | A | Intronic | Modifier | - | 2 | c.1084-946G>A | - | rs114456709 |
| 29880787 | G | A | Intronic | Modifier | - | 2 | c.1084-925G>A | - | - |
| 29880800 | G | A | Intronic | Modifier | - | 2 | c.1084-912G>A | - | rs1010587199 |
| 29880804 | G | C | Intronic | Modifier | - | 2 | c.1084-908G>C | - | rs573878341 |
| 29880814 | C | T | Intronic | Modifier | - | 2 | c.1084-898C>T | - | rs868582729 |
| 29880823 | T | G | Intronic | Modifier | - | 2 | c.1084-889T>G | - | - |
| 29880856 | C | T | Intronic | Modifier | - | 2 | c.1084-856C>T | - | rs1366171508 |
| 29880884 | C | G | Intronic | Modifier | - | 2 | c.1084-828C>G | - | rs566334659 |
| 29880907 | T | C | Intronic | Modifier | - | 2 | c.1084-805T>C | - | rs931732579 |
| 29880914 | T | C | Intronic | Modifier | - | 2 | c.1084-798T>C | - | rs5763264 |
| 29880916 | C | T | Intronic | Modifier | - | 2 | c.1084-796C>T | - | rs553558060 |
| 29880942 | G | A | Intronic | Modifier | - | 2 | c.1084-770G>A | - | - |
| 29880948 | T | G | Intronic | Modifier | - | 2 | c.1084-764T>G | - | - |
| 29880977 | C | T | Intronic | Modifier | - | 2 | c.1084-735C>T | - | rs1181856343 |
| 29880990 | A | C | Intronic | Modifier | - | 2 | c.1084-722A>C | - | - |
| 29881010 | A | G | Intronic | Modifier | - | 2 | c.1084-702A>G | - | rs1319767449 |
| 29881149 | T | C | Intronic | Modifier | - | 2 | c.1084-563T>C | - | - |
| 29881150 | G | GTAT | Intronic | Modifier | - | 2 | c.1084-560_1084-557dup | - | rs1286429529 |
| 29881162 | G | A | Intronic | Modifier | - | 2 | c.1084-550G>A | - | - |
| 29881176 | C | T | Intronic | Modifier | - | 2 | c.1084-536C>T | - | rs559654353 |
| 29881195 | A | G | Intronic | Modifier | - | 2 | c.1084-517A>G | - | rs913417474 |
| 29881212 | C | T | Intronic | Modifier | - | 2 | c.1084-500C>T | - | - |
| 29881214 | C | T | Intronic | Modifier | - | 2 | c.1084-498C>T | - | rs2240635 |
| 29881237 | A | G | Intronic | Modifier | - | 2 | c.1084-475A>G | - | rs142119901 |
| 29881265 | T | C | Intronic | Modifier | - | 2 | c.1084-447T>C | - | rs1040695377 |
| 29881277 | A | G | Intronic | Modifier | - | 2 | c.1084-435A>G | - | - |
| 29881292 | A | T | Intronic | Modifier | - | 2 | c.1084-420A>T | - | - |
| 29881333 | C | G | Intronic | Modifier | - | 2 | c.1084-379C>G | - | - |
| 29881340 | C | T | Intronic | Modifier | - | 2 | c.1084-372C>T | - | rs1303062643 |
| 29881369 | G | A | Intronic | Modifier | - | 2 | c.1084-343G>A | - | rs534920944 |
| 29881378 | C | T | Intronic | Modifier | - | 2 | c.1084-334C>T | - | rs555490349 |
| 29881388 | G | A | Intronic | Modifier | - | 2 | c.1084-324G>A | - | rs138583539 |
| 29881405 | T | C | Intronic | Modifier | - | 2 | c.1084-307T>C | - | - |
| 29881459 | G | A | Intronic | Modifier | - | 2 | c.1084-253G>A | - | rs946441565 |
| 29881461 | A | G | Intronic | Modifier | - | 2 | c.1084-251A>G | - | rs12484008 |
| 29881468 | G | A | Intronic | Modifier | - | 2 | c.1084-244G>A | - | rs3815335 |
| 29881537 | C | T | Intronic | Modifier | - | 2 | c.1084-175C>T | - | rs78248741 |
| 29881559 | A | G | Intronic | Modifier | - | 2 | c.1084-153A>G | - | - |
| 29881622 | G | A | Intronic | Modifier | - | 2 | c.1084-90G>A | - | rs368599359 |
| 29881633 | G | A | Intronic | Modifier | - | 2 | c.1084-79G>A | - | rs533283887 |
| 29881645 | G | A | Intronic | Modifier | - | 2 | c.1084-67G>A | - | rs551382175 |
| 29881673 | A | G | Intronic | Modifier | - | 2 | c.1084-39A>G | - | rs755841191 |
| 29881683 | A | G | Intronic | Modifier | - | 2 | c.1084-29A>G | - | - |
| 29881703 | C | T | Intronic | Low | - | 2 | c.1084-9C>T | - | - |
| 29881704 | C | G | Intronic | Low | - | 2 | c.1084-8C>G | - | - |
| 29881727 | C | T | Synonymous | Low | 3 | - | c.1099C>T | p.Leu367Leu | - |
| 29881776 | G | A | Missense | Moderate | 3 | - | c.1148G>A | p.Arg383Gln | rs377149236 |
| 29881797 | A | C | Missense | Moderate | 3 | - | c.1169A>C | p.Asn390Thr | rs148653339 |
| 29881810 | T | G | Synonymous | Low | 3 | - | c.1182T>G | p.Ala394Ala | rs1033200120 |
| 29881828 | C | T | Synonymous | Low | 3 | - | c.1200C>T | p.Ala400Ala | rs165734 |
| 29881854 | T | C | Intronic | Modifier | - | 3 | c.1208+18T>C | - | - |
| 29881868 | G | A | Intronic | Modifier | - | 3 | c.1208+32G>A | - | - |
| 29881884 | G | A | Intronic | Modifier | - | 3 | c.1208+48G>A | - | rs4823040 |
| 29881885 | C | T | Intronic | Modifier | - | 3 | c.1208+49C>T | - | rs540168753 |
| 29881890 | C | T | Intronic | Modifier | - | 3 | c.1208+54C>T | - | rs114903253 |
| 29881907 | C | G | Intronic | Modifier | - | 3 | c.1208+71C>G | - | rs1187543862 |
| 29881962 | C | T | Intronic | Modifier | - | 3 | c.1208+126C>T | - | rs1031194111 |
| 29881963 | G | A | Intronic | Modifier | - | 3 | c.1208+127G>A | - | rs937910485 |
| 29881990 | C | T | Intronic | Modifier | - | 3 | c.1208+154C>T | - | rs1466417517 |
| 29882005 | C | A | Intronic | Modifier | - | 3 | c.1208+169C>A | - | rs1056424058 |
| 29882071 | G | A | Intronic | Modifier | - | 3 | c.1208+235G>A | - | rs779090983 |
| 29882082 | A | G | Intronic | Modifier | - | 3 | c.1208+246A>G | - | - |
| 29882092 | C | T | Intronic | Modifier | - | 3 | c.1208+256C>T | - | rs897689341 |
| 29882110 | C | T | Intronic | Modifier | - | 3 | c.1208+274C>T | - | rs1012266468 |
| 29882111 | G | A | Intronic | Modifier | - | 3 | c.1208+275G>A | - | rs28525662 |
| 29882150 | C | T | Intronic | Modifier | - | 3 | c.1208+314C>T | - | rs1177767322 |
| 29882157 | G | A | Intronic | Modifier | - | 3 | c.1208+321G>A | - | rs147136515 |
| 29882183 | T | A | Intronic | Modifier | - | 3 | c.1208+347_1208+348insA | - | rs111690803 |
| 29882184 | T | A | Intronic | Modifier | - | 3 | c.1208+348T>A | - | rs190911251 |
| 29882187 | T | A | Intronic | Modifier | - | 3 | c.1208+351T>A | - | rs375863529 |
| 29882247 | C | G | Intronic | Modifier | - | 3 | c.1208+411C>G | - | rs143316545 |
| 29882294 | G | A | Intronic | Modifier | - | 3 | c.1208+458G>A | - | rs5997481 |
| 29882303 | C | T | Intronic | Modifier | - | 3 | c.1208+467C>T | - | - |
| 29882324 | A | G | Intronic | Modifier | - | 3 | c.1208+488A>G | - | rs6006162 |
| 29882326 | G | C | Intronic | Modifier | - | 3 | c.1208+490G>C | - | rs903889822 |
| 29882328 | T | A | Intronic | Modifier | - | 3 | c.1208+492T>A | - | - |
| 29882386 | G | C | Intronic | Modifier | - | 3 | c.1208+550G>C | - | - |
| 29882403 | T | C | Intronic | Modifier | - | 3 | c.1208+567T>C | - | rs1319062352 |
| 29882443 | G | A | Intronic | Modifier | - | 3 | c.1208+607G>A | - | rs188013725 |
| 29882460 | C | T | Intronic | Modifier | - | 3 | c.1208+624C>T | - | rs555368787 |
| 29882484 | T | A | Intronic | Modifier | - | 3 | c.1208+648T>A | - | - |
| 29882489 | T | A | Intronic | Modifier | - | 3 | c.1208+653T>A | - | rs752352485 |
| 29882500 | A | T | Intronic | Modifier | - | 3 | c.1208+664A>T | - | rs1240145238 |
| 29882506 | G | T | Intronic | Modifier | - | 3 | c.1208+671dup | - | - |
| 29882508 | TC | - | Intronic | Modifier | - | 3 | c.1208+672del | - | rs527873265 |
| 29882530 | TTTTG | - | Intronic | Modifier | - | 3 | c.1208+691_1208+694del | - | rs1198608745 |
| 29882546 | C | T | Intronic | Modifier | - | 3 | c.1208+710C>T | - | rs757583792 |
| 29882555 | A | T | Intronic | Modifier | - | 3 | c.1208+719A>T | - | rs193062610 |
| 29882556 | G | A | Intronic | Modifier | - | 3 | c.1208+720G>A | - | rs963017814 |
| 29882569 | C | T | Intronic | Modifier | - | 3 | c.1208+733C>T | - | rs1231911858 |
| 29882571 | AG | - | Intronic | Modifier | - | 3 | c.1208+735del | - | - |
| 29882573 | A | C | Intronic | Modifier | - | 3 | c.1208+737A>C | - | rs780861559 |
| 29882580 | C | T | Intronic | Modifier | - | 3 | c.1208+744C>T | - | rs1392375542 |
| 29882630 | G | A | Intronic | Modifier | - | 3 | c.1208+794G>A | - | rs747407223 |
| 29882664 | A | G | Intronic | Modifier | - | 3 | c.1208+828A>G | - | - |
| 29882687 | T | A | Intronic | Modifier | - | 3 | c.1208+851T>A | - | rs150888215 |
| 29882693 | G | A | Intronic | Modifier | - | 3 | c.1208+857G>A | - | rs531344127 |
| 29882694 | T | G | Intronic | Modifier | - | 3 | c.1208+858T>G | - | - |
| 29882713 | T | C | Intronic | Modifier | - | 3 | c.1208+877T>C | - | - |
| 29882715 | T | C | Intronic | Modifier | - | 3 | c.1208+879T>C | - | - |
| 29882738 | C | T | Intronic | Modifier | - | 3 | c.1208+902C>T | - | - |
| 29882754 | C | T | Intronic | Modifier | - | 3 | c.1208+918C>T | - | rs1051929078 |
| 29882755 | G | A | Intronic | Modifier | - | 3 | c.1208+919G>A | - | rs139362410 |
| 29882760 | C | T | Intronic | Modifier | - | 3 | c.1208+924C>T | - | rs551722146 |
| 29882791 | C | A | Intronic | Modifier | - | 3 | c.1208+955C>A | - | rs537125677 |
| 29882794 | G | A | Intronic | Modifier | - | 3 | c.1208+958G>A | - | rs1026424254 |
| 29882813 | A | G | Intronic | Modifier | - | 3 | c.1208+977A>G | - | rs1299918179 |
| 29882830 | C | T | Intronic | Modifier | - | 3 | c.1208+994C>T | - | rs556908766 |
| 29882838 | C | A | Intronic | Modifier | - | 3 | c.1208+1002C>A | - | rs187342281 |
| 29882849 | G | T | Intronic | Modifier | - | 3 | c.1208+1013G>T | - | rs980830606 |
| 29882850 | A | C | Intronic | Modifier | - | 3 | c.1208+1014A>C | - | rs925373768 |
| 29882857 | C | T | Intronic | Modifier | - | 3 | c.1208+1021C>T | - | rs936782201 |
| 29882864 | A | G | Intronic | Modifier | - | 3 | c.1208+1028A>G | - | rs913923260 |
| 29882865 | G | A | Intronic | Modifier | - | 3 | c.1208+1029G>A | - | rs1205303005 |
| 29882882 | C | T | Intronic | Modifier | - | 3 | c.1208+1046C>T | - | rs764790044 |
| 29882902 | C | T | Intronic | Modifier | - | 3 | c.1208+1066C>T | - | - |
| 29882915 | G | C | Intronic | Modifier | - | 3 | c.1208+1079G>C | - | rs1486110150 |
| 29882945 | A | G | Intronic | Modifier | - | 3 | c.1208+1109A>G | - | rs150023132 |
| 29882967 | T | G | Intronic | Modifier | - | 3 | c.1208+1131T>G | - | - |
| 29882973 | C | T | Intronic | Modifier | - | 3 | c.1208+1137C>T | - | rs165899 |
| 29882976 | C | G | Intronic | Modifier | - | 3 | c.1208+1140C>G | - | rs542818912 |
| 29883007 | A | T | Intronic | Modifier | - | 3 | c.1208+1171A>T | - | - |
| 29883018 | A | G | Intronic | Modifier | - | 3 | c.1208+1182A>G | - | rs573136532 |
| 29883076 | C | T | Intronic | Modifier | - | 3 | c.1208+1240C>T | - | rs553734179 |
| 29883087 | A | T | Intronic | Modifier | - | 3 | c.1208+1251A>T | - | rs1304338716 |
| 29883094 | CA | - | Intronic | Modifier | - | 3 | c.1208+1258del | - | - |
| 29883123 | TAGTC | - | Intronic | Modifier | - | 3 | c.1208+1287_1208+1290del | - | rs362061 |
| 29883162 | C | G | Intronic | Modifier | - | 3 | c.1208+1326C>G | - | - |
| 29883164 | G | T | Intronic | Modifier | - | 3 | c.1208+1328G>T | - | rs561170932 |
| 29883165 | G | C | Intronic | Modifier | - | 3 | c.1208+1329G>C | - | - |
| 29883208 | T | C | Intronic | Modifier | - | 3 | c.1208+1372T>C | - | rs1201391625 |
| 29883254 | G | T | Intronic | Modifier | - | 3 | c.1208+1418G>T | - | - |
| 29883316 | C | G | Intronic | Modifier | - | 3 | c.1208+1480C>G | - | - |
| 29883346 | G | A | Intronic | Modifier | - | 3 | c.1209-1492G>A | - | rs189670956 |
| 29883380 | G | A | Intronic | Modifier | - | 3 | c.1209-1458G>A | - | - |
| 29883413 | G | C | Intronic | Modifier | - | 3 | c.1209-1425G>C | - | - |
| 29883466 | A | AAG | Intronic | Modifier | - | 3 | c.1209-1370_1209-1369insGAA | - | rs1285972523 |
| 29883513 | C | T | Intronic | Modifier | - | 3 | c.1209-1325C>T | - | rs1030352345 |
| 29883524 | C | T | Intronic | Modifier | - | 3 | c.1209-1314C>T | - | rs750190295 |
| 29883525 | G | A | Intronic | Modifier | - | 3 | c.1209-1313G>A | - | rs950766752 |
| 29883530 | A | T | Intronic | Modifier | - | 3 | c.1209-1308A>T | - | - |
| 29883561 | C | T | Intronic | Modifier | - | 3 | c.1209-1277C>T | - | - |
| 29883568 | C | T | Intronic | Modifier | - | 3 | c.1209-1270C>T | - | - |
| 29883576 | G | A | Intronic | Modifier | - | 3 | c.1209-1262G>A | - | rs373486863 |
| 29883630 | TAAC | - | Intronic | Modifier | - | 3 | c.1209-1203_1209-1201del | - | rs916778818 |
| 29883635 | A | C | Intronic | Modifier | - | 3 | c.1209-1203A>C | - | rs1418123505 |
| 29883636 | C | A | Intronic | Modifier | - | 3 | c.1209-1202C>A | - | rs1164703398 |
| 29883648 | A | C | Intronic | Modifier | - | 3 | c.1209-1190A>C | - | - |
| 29883658 | C | T | Intronic | Modifier | - | 3 | c.1209-1180C>T | - | rs1281070945 |
| 29883659 | G | A | Intronic | Modifier | - | 3 | c.1209-1179G>A | - | rs143625327 |
| 29883662 | C | T | Intronic | Modifier | - | 3 | c.1209-1176C>T | - | rs1334347932 |
| 29883710 | C | G | Intronic | Modifier | - | 3 | c.1209-1128C>G | - | - |
| 29883714 | G | A | Intronic | Modifier | - | 3 | c.1209-1124G>A | - | rs998962613 |
| 29883772 | CACTT | - | Intronic | Modifier | - | 3 | c.1209-1066_1209-1063del | - | rs362231 |
| 29883787 | A | G | Intronic | Modifier | - | 3 | c.1209-1051A>G | - | rs901063529 |
| 29883797 | G | A | Intronic | Modifier | - | 3 | c.1209-1041G>A | - | rs571615682 |
| 29883810 | A | C | Intronic | Modifier | - | 3 | c.1209-1028A>C | - | - |
| 29883836 | C | T | Intronic | Modifier | - | 3 | c.1209-1002C>T | - | rs781731242 |
| 29883842 | G | T | Intronic | Modifier | - | 3 | c.1209-996G>T | - | rs563602777 |
| 29883892 | G | T | Intronic | Modifier | - | 3 | c.1209-946G>T | - | rs174650 |
| 29883923 | G | A | Intronic | Modifier | - | 3 | c.1209-915G>A | - | - |
| 29883942 | A | T | Intronic | Modifier | - | 3 | c.1209-896A>T | - | - |
| 29883945 | A | C | Intronic | Modifier | - | 3 | c.1209-893A>C | - | - |
| 29883958 | A | C | Intronic | Modifier | - | 3 | c.1209-880A>C | - | rs80091782 |
| 29884011 | C | T | Intronic | Modifier | - | 3 | c.1209-827C>T | - | rs555103444 |
| 29884027 | G | A | Intronic | Modifier | - | 3 | c.1209-811G>A | - | rs769503095 |
| 29884037 | C | T | Intronic | Modifier | - | 3 | c.1209-801C>T | - | - |
| 29884047 | C | T | Intronic | Modifier | - | 3 | c.1209-791C>T | - | - |
| 29884073 | C | T | Intronic | Modifier | - | 3 | c.1209-765C>T | - | rs141161567 |
| 29884123 | G | A | Intronic | Modifier | - | 3 | c.1209-715G>A | - | rs566071826 |
| 29884131 | C | T | Intronic | Modifier | - | 3 | c.1209-707C>T | - | rs528754360 |
| 29884181 | C | T | Intronic | Modifier | - | 3 | c.1209-657C>T | - | rs567502217 |
| 29884182 | G | A | Intronic | Modifier | - | 3 | c.1209-656G>A | - | rs185791469 |
| 29884208 | C | T | Intronic | Modifier | - | 3 | c.1209-630C>T | - | rs377340059 |
| 29884226 | G | A | Intronic | Modifier | - | 3 | c.1209-612G>A | - | rs1432228295 |
| 29884229 | CGTGCGCCT | - | Intronic | Modifier | - | 3 | c.1209-614_1209-607del | - | - |
| 29884231 | T | C | Intronic | Modifier | - | 3 | c.1209-607T>C | - | rs956995883 |
| 29884235 | C | T | Intronic | Modifier | - | 3 | c.1209-603C>T | - | rs971572887 |
| 29884278 | G | A | Intronic | Modifier | - | 3 | c.1209-560G>A | - | rs935759554 |
| 29884285 | T | G | Intronic | Modifier | - | 3 | c.1209-553T>G | - | rs571203555 |
| 29884367 | G | A | Intronic | Modifier | - | 3 | c.1209-471G>A | - | rs536728781 |
| 29884432 | T | C | Intronic | Modifier | - | 3 | c.1209-406T>C | - | - |
| 29884485 | T | C | Intronic | Modifier | - | 3 | c.1209-353T>C | - | rs6006163 |
| 29884509 | G | T | Intronic | Modifier | - | 3 | c.1209-329G>T | - | rs760318118 |
| 29884520 | T | C | Intronic | Modifier | - | 3 | c.1209-318T>C | - | rs886441058 |
| 29884567 | C | T | Intronic | Modifier | - | 3 | c.1209-271C>T | - | rs165624 |
| 29884574 | A | G | Intronic | Modifier | - | 3 | c.1209-264A>G | - | - |
| 29884575 | G | T | Intronic | Modifier | - | 3 | c.1209-263G>T | - | rs181661746 |
| 29884599 | C | T | Intronic | Modifier | - | 3 | c.1209-239C>T | - | - |
| 29884603 | A | G | Intronic | Modifier | - | 3 | c.1209-235A>G | - | rs557043300 |
| 29884612 | A | T | Intronic | Modifier | - | 3 | c.1209-226A>T | - | rs1022377385 |
| 29884639 | C | T | Intronic | Modifier | - | 3 | c.1209-199C>T | - | rs541500423 |
| 29884640 | G | A | Intronic | Modifier | - | 3 | c.1209-198G>A | - | rs912855297 |
| 29884643 | G | T | Intronic | Modifier | - | 3 | c.1209-195G>T | - | rs751682286 |
| 29884650 | T | C | Intronic | Modifier | - | 3 | c.1209-188T>C | - | rs982583256 |
| 29884712 | T | G | Intronic | Modifier | - | 3 | c.1209-126T>G | - | rs1323270119 |
| 29884734 | C | T | Intronic | Modifier | - | 3 | c.1209-104C>T | - | rs565596168 |
| 29884736 | C | G | Intronic | Modifier | - | 3 | c.1209-102C>G | - | rs1039501899 |
| 29884799 | C | T | Intronic | Modifier | - | 3 | c.1209-39C>T | - | rs376487122 |
| 29884804 | GT | - | Intronic | Modifier | - | 3 | c.1209-32del | - | rs1427581204 |
| 29884809 | A | G | Intronic | Modifier | - | 3 | c.1209-29A>G | - | rs746250651 |
| 29884814 | T | C | Intronic | Modifier | - | 3 | c.1209-24T>C | - | rs770018275 |
| 29884817 | G | A | Intronic | Modifier | - | 3 | c.1209-21G>A | - | rs201134753 |
| 29884869 | G | A | Missense | Moderate | 4 | - | c.1240G>A | p.Gly414Ser | - |
| 29884881 | A | G | Missense | Moderate | 4 | - | c.1252A>G | p.Ile418Val | rs751075628 |
| 29884939 | A | G | Missense | Moderate | 4 | - | c.1310A>G | p.Lys437Arg | rs1602971285 |
| 29884940 | G | T | Missense | Moderate | 4 | - | c.1311G>T | p.Lys437Asn | - |
| 29884950 | G | A | Missense | Moderate | 4 | - | c.1321G>A | p.Glu441Lys | rs145061116 |
| 29884976 | G | C | Missense | Moderate | 4 | - | c.1347G>C | p.Lys449Asn | rs199932977 |
| 29885016 | G | A | Missense | Moderate | 4 | - | c.1387G>A | p.Glu463Lys | rs59371099 |
| 29885058 | G | C | Missense | Moderate | 4 | - | c.1429G>C | p.Glu477Gln | - |
| 29885075 | G | A | Synonymous | Low | 4 | - | c.1446G>A | p.Glu482Glu | rs5997482 |
| 29885120 | TGAAGAAGAGGAGGCAGAAGGGGGA | - | In-frame Deletion | Moderate | 4 | - | c.1476_1499del | p.Glu493_Glu500del | rs748774766 |
| 29885127 | G | AAACAA | In-frame Insertion | Moderate | 4 | - | c.1500_1505dup | p.Thr501_Lys502dup | rs576586746 |
| 29885164 | C | G | Missense | Moderate | 4 | - | c.1535C>G | p.Pro512Arg | - |
| 29885198 | G | C | Missense | Moderate | 4 | - | c.1569G>C | p.Glu523Asp | rs138278265 |
| 29885202 | A | G | Missense | Moderate | 4 | - | c.1573A>G | p.Lys525Glu | rs751816018 |
| 29885204 | G | C | Missense | Moderate | 4 | - | c.1575G>C | p.Lys525Asn | rs149183166 |
| 29885255 | C | T | Synonymous | Low | 4 | - | c.1626C>T | p.Ala542Ala | rs747901802 |
| 29885260 | CCAAGTCCCCAGAGAAGGAGGAAGCAAAATCCCCAGCCGAAGT | - | In-frame Deletion | Moderate | 4 | - | c.1599_1640del | p.Glu536_Lys549del | rs1353196821 |
| 29885308 | C | T | Missense | Moderate | 4 | - | c.1679C>T | p.Ser560Leu | rs750569231 |
| 29885311 | C | T | Missense | Moderate | 4 | - | c.1682C>T | p.Pro561Leu | rs548556825 |
| 29885313 | C | G | Missense | Moderate | 4 | - | c.1684C>G | p.Pro562Ala | rs530872313 |
| 29885369 | C | T | Synonymous | Low | 4 | - | c.1740C>T | p.Ser580Ser | rs114263951 |
| 29885378 | G | GAA | In-frame Insertion | Moderate | 4 | - | c.1750_1751insAAG | p.Lys583_Ala584insGlu | - |
| 29885383 | A | G | Missense | Moderate | 4 | - | c.1754A>G | p.Lys585Arg | - |
| 29885386 | C | T | Missense | Moderate | 4 | - | c.1757C>T | p.Ser586Phe | - |
| 29885403 | G | T | Missense | Moderate | 4 | - | c.1774G>T | p.Ala592Ser | rs768529407 |
| 29885412 | C | T | Missense | Moderate | 4 | - | c.1783C>T | p.Pro595Ser | rs6006165 |
| 29885423 | AAAGTCACCGGCTGAGGCC | - | In-frame Deletion | Moderate | 4 | - | c.1782_1799del | p.Ala596_Pro601del | - |
| 29885425 | A | C | Missense | Moderate | 4 | - | c.1796A>C | p.Lys599Thr | rs1403224343 |
| 29885436 | A | AGGCCAAGTCCCCAGCGAAGGAAGAAGCAAAGTCCCCGGCTG | In-frame Insertion | Moderate | 4 | - | c.1822_1823insCGAAGGAAGAAGCAAAGTCCCCGGCTGAGGCCAAGTCCCCAG | p.Pro607_Val608insAlaLysGluGluAlaLysSerProAlaGluAlaLysSerPro | - |
| 29885438 | G | GAA | In-frame Insertion | Moderate | 4 | - | c.1810_1811insAAG | p.Lys603_Ala604insGlu | - |
| 29885462 | A | G | Synonymous | Low | 4 | - | c.1833A>G | p.Glu611Glu | rs1196681707 |
| 29885475 | G | C | Missense | Moderate | 4 | - | c.1846G>C | p.Ala616Pro | rs1398309477 |
| 29885508 | A | C | Missense | Moderate | 4 | - | c.1879A>C | p.Lys627Gln | rs1353842666 |
| 29885554 | C | T | Missense | Moderate | 4 | - | c.1925C>T | p.Thr642Met | rs117258406 |
| 29885556 | CCAGCTGAGGTCAAGTCCCCGGAAAAGGCCAAGTCTCCAACGA | - | In-frame Deletion | Moderate | 4 | - | c.1886_1927del | p.Pro629_Lys643delinsGln | - |
| 29885558 | AGCTGAGGTCAAGTCCCCGGAAAAGGCCAAGTCTCCAACGAAG | - | In-frame Deletion | Moderate | 4 | - | c.1889_1930del | p.Ala630_Lys643del | - |
| 29885564 | GGTCAAGTCCCCGGAAAAGGCCAAGTCTCCAACGAAGGAGGAA | - | In-frame Deletion | Moderate | 4 | - | c.1895_1936del | p.Val632_Glu645del | - |
| 29885564 | A | GCAAAGTCCCCTGAGAAG | In-frame Insertion | Moderate | 4 | - | c.1938_1955dup | p.Ala652_Lys657dup | - |
| 29885570 | G | C | Missense | Moderate | 4 | - | c.1941G>C | p.Lys647Asn | rs200634512 |
| 29885571 | T | CCCCTGAGAAGGCCAAGG | In-frame Insertion | Moderate | 4 | - | c.1959_1960insGCCCCTGAGAAGGCCAAG | p.Lys653_Ser654insAlaProGluLysAlaLys | - |
| 29885576 | GGAAAAGGCCAAGTCTCCAACGAAGGAGGAAGCAAAGTCCCCT | - | In-frame Deletion | Moderate | 4 | - | c.1908_1949del | p.Thr642_Pro655del | - |
| 29885582 | G | GCCAAGTCCCCTGAGAAC | In-frame Insertion | Moderate | 4 | - | c.1964_1965insTGAGAACGCCAAGTCCCC | p.Glu656_Lys657insAsnAlaLysSerProGlu | - |
| 29885585 | C | AAGGCCCCAGAGAAGGCA | In-frame Insertion | Moderate | 4 | - | c.1959_1960insGCCCCAGAGAAGGCAAAG | p.Lys653_Ser654insAlaProGluLysAlaLys | - |
| 29885599 | A | GGACAAGGCCCCAGCGAC | In-frame Insertion | Moderate | 4 | - | c.1973_1974insCAAGGCCCCAGCGACGGA | p.Lys657_Glu658insAspLysAlaProAlaThr | - |
| 29885601 | G | CC | Frameshift Insertion | High | 4 | - | c.1972_1973insCC | p.Glu658AlafsTer14 | rs1427435154 |
| 29885604 | AAGGCCAAGTCCCCAGAGAAGGAAG | - | In-frame Deletion | Moderate | 4 | - | c.1965_1988del | p.Glu658_Lys665del | rs267607533 |
| 29885604 | AAGGAAG | - | In-frame Deletion | Moderate | 4 | - | c.1973_1978del | p.Glu658_Glu659del | rs149571560 |
| 29885622 | GAGGCCAAGTCCCCTGAGA | - | In-frame Deletion | Moderate | 4 | - | c.1989_2006del | p.Glu664_Pro669del | rs267607534 |
| 29885622 | A | AGGGAG | In-frame Insertion | Moderate | 4 | - | c.1996_1997insGAGAGG | p.Lys665_Ala666insGlyGlu | - |
| 29885640 | A | C | Missense | Moderate | 4 | - | c.2011A>C | p.Lys671Gln | - |
| 29885648 | A | G | Synonymous | Low | 4 | - | c.2019A>G | p.Glu673Glu | rs746048017 |
| 29885651 | A | C | Synonymous | Low | 4 | - | c.2022A>C | p.Ala674Ala | rs763507482 |
| 29885711 | C | T | Synonymous | Low | 4 | - | c.2082C>T | p.Ala694Ala | rs1452419487 |
| 29885736 | A | C | Missense | Moderate | 4 | - | c.2107A>C | p.Lys703Gln | - |
| 29885742 | C | T | Missense | Moderate | 4 | - | c.2113C>T | p.Pro705Ser | rs770039871 |
| 29885783 | C | T | Synonymous | Low | 4 | - | c.2154C>T | p.Ser718Ser | rs772385634 |
| 29885819 | AAAGACCCCCGAGAAGGCCAAGTCCCCAGTGAAGGAAGAAGCT | C | In-frame Deletion | Moderate | 4 | - | c.2190_2232delinsC | p.Thr732_Lys745del | - |
| 29885835 | G | T | Missense | Moderate | 4 | - | c.2206G>T | p.Ala736Ser | - |
| 29885861 | AAAGACCCCCGAGAAGGCCAAGTCCCCAGTGAAGGAAGAAGCT | C | In-frame Deletion | Moderate | 4 | - | c.2232_2274delinsC | p.Lys745_Ala758del | - |
| 29885861 | CAAGTCCCCAGTGAAGGAAGAAGCT | - | In-frame Deletion | Moderate | 4 | - | c.2219_2242del | p.Val740_Pro747del | rs778386609 |
| 29885870 | CGAGAAGGCCAAGTCCCCAGTGAAGGAAGAAGCTAAGTCCCCA | - | In-frame Deletion | Moderate | 4 | - | c.2219_2260del | p.Val740_Pro753del | - |
| 29885884 | C | T | Missense | Moderate | 4 | - | c.2255C>T | p.Ser752Phe | - |
| 29885913 | A | G | Missense | Moderate | 4 | - | c.2284A>G | p.Lys762Glu | - |
| 29885939 | G | A | Synonymous | Low | 4 | - | c.2310G>A | p.Ala770Ala | rs757096652 |
| 29885959 | C | T | Missense | Moderate | 4 | - | c.2330C>T | p.Pro777Leu | rs199748453 |
| 29885976 | G | A | Missense | Moderate | 4 | - | c.2347G>A | p.Glu783Lys | - |
| 29885996 | CAAG | - | In-frame Deletion | Moderate | 4 | - | c.2368_2370del | p.Lys790del | rs59551486 |
| 29886001 | A | C | Missense | Moderate | 4 | - | c.2372A>C | p.Glu791Ala | - |
| 29886025 | C | T | Missense | Moderate | 4 | - | c.2396C>T | p.Ala799Val | rs375529650 |
| 29886026 | G | A | Synonymous | Low | 4 | - | c.2397G>A | p.Ala799Ala | rs376035598 |
| 29886034 | C | T | Missense | Moderate | 4 | - | c.2405C>T | p.Pro802Leu | rs758520448 |
| 29886043 | A | C | Missense | Moderate | 4 | - | c.2414A>C | p.Glu805Ala | rs165602 |
| 29886044 | G | A | Synonymous | Low | 4 | - | c.2415G>A | p.Glu805Glu | rs940285527 |
| 29886069 | A | G | Missense | Moderate | 4 | - | c.2440A>G | p.Ile814Val | rs748832999 |
| 29886072 | C | A | Missense | Moderate | 4 | - | c.2443C>A | p.Pro815Thr | - |
| 29886088 | T | C | Missense | Moderate | 4 | - | c.2459T>C | p.Val820Ala | - |
| 29886140 | G | A | Synonymous | Low | 4 | - | c.2511G>A | p.Glu837Glu | rs751566708 |
| 29886147 | A | G | Missense | Moderate | 4 | - | c.2518A>G | p.Lys840Glu | rs750908593 |
| 29886189 | CAG | - | Frameshift Variant | High | 4 | - | c.2561_2562del | p.Glu854GlyfsTer37 | rs775700827 |
| 29886200 | G | A | Synonymous | Low | 4 | - | c.2571G>A | p.Lys857Lys | rs145669735 |
| 29886230 | G | T | Missense | Moderate | 4 | - | c.2601G>T | p.Lys867Asn | rs138156220 |
| 29886253 | TGGA | - | In-frame Deletion | Moderate | 4 | - | c.2626_2628del | p.Glu876del | rs768937935 |
| 29886268 | C | T | Missense | Moderate | 4 | - | c.2639C>T | p.Pro880Leu | rs756683695 |
| 29886275 | C | T | Synonymous | Low | 4 | - | c.2646C>T | p.Val882Val | rs528790943 |
| 29886276 | G | A | Missense | Moderate | 4 | - | c.2647G>A | p.Glu883Lys | rs754121553 |
| 29886289 | A | G | Missense | Moderate | 4 | - | c.2660A>G | p.Glu887Gly | - |
| 29886352 | A | G | Missense | Moderate | 4 | - | c.2723A>G | p.Lys908Arg | - |
| 29886360 | C | T | Missense | Moderate | 4 | - | c.2731C>T | p.Pro911Ser | rs376751999 |
| 29886375 | G | A | Missense | Moderate | 4 | - | c.2746G>A | p.Val916Met | rs762057603 |
| 29886386 | C | T | Synonymous | Low | 4 | - | c.2757C>T | p.Asp919Asp | rs56200920 |
| 29886400 | A | C | Missense | Moderate | 4 | - | c.2771A>C | p.Glu924Ala | rs199737170 |
| 29886413 | A | G | Synonymous | Low | 4 | - | c.2784A>G | p.Val928Val | rs165625 |
| 29886415 | C | A | Missense | Moderate | 4 | - | c.2786C>A | p.Ala929Asp | - |
| 29886478 | C | T | Missense | Moderate | 4 | - | c.2849C>T | p.Ala950Val | rs553629461 |
| 29886485 | G | A | Synonymous | Low | 4 | - | c.2856G>A | p.Pro952Pro | rs147416824 |
| 29886498 | A | G | Missense | Moderate | 4 | - | c.2869A>G | p.Thr957Ala | rs760693353 |
| 29886509 | G | T | Missense | Moderate | 4 | - | c.2880G>T | p.Glu960Asp | rs372070551 |
| 29886517 | A | G | Missense | Moderate | 4 | - | c.2888A>G | p.Lys963Arg | rs1480895257 |
| 29886543 | CAG | - | Frameshift Variant | High | 4 | - | c.2915_2916del | p.Glu972GlyfsTer7 | rs1346887118 |
| 29886635 | A | G | Synonymous | Low | 4 | - | c.3006A>G | p.Gln1002Gln | rs768522862 |
| 29886673 | A | G | Missense | Moderate | 4 | - | c.3044A>G | p.Lys1015Arg | - |
| 29886713 | C | T | 3' UTR | Modifier | 4 | - | c.*21C>T |  | rs191972471 |
| 29886714 | G | A | 3' UTR | Modifier | 4 | - | c.*22G>A |  | rs183499750 |
| 29886718 | C | G | 3' UTR | Modifier | 4 | - | c.*26C>G |  | rs374202245 |
| 29886726 | G | A | 3' UTR | Modifier | 4 | - | c.*34G>A |  | - |
| 29886744 | G | A | 3' UTR | Modifier | 4 | - | c.*52G>A |  | rs766995345 |
| 29886758 | A | G | 3' UTR | Modifier | 4 | - | c.*66A>G |  | rs753770865 |
| 29886778 | T | C | 3' UTR | Modifier | 4 | - | c.*86T>C |  | rs1270308635 |
| 29886797 | GA | - | 3' UTR | Modifier | 4 | - | c.*106del |  | rs748195897 |
| 29886798 | A | G | 3' UTR | Modifier | 4 | - | c.*106A>G |  | rs771159816 |
| 29886815 | C | T | 3' UTR | Modifier | 4 | - | c.*123C>T |  | rs781167046 |
| 29886835 | A | G | 3' UTR | Modifier | 4 | - | c.*143A>G |  | rs1050302661 |
| 29886840 | AT | - | 3' UTR | Modifier | 4 | - | c.*150del |  | rs747501203 |
| 29886852 | A | G | 3' UTR | Modifier | 4 | - | c.*160A>G |  | - |
| 29886893 | G | T | 3' UTR | Modifier | 4 | - | c.*201G>T |  | rs1061373 |
| 29886906 | C | T | 3' UTR | Modifier | 4 | - | c.*214C>T |  | rs533220152 |
| 29886907 | G | A | 3' UTR | Modifier | 4 | - | c.*215G>A |  | rs755046912 |
| 29886909 | T | C | 3' UTR | Modifier | 4 | - | c.*217T>C |  | rs897216138 |
| 29887012 | T | C | 3' UTR | Modifier | 4 | - | c.*320T>C |  | rs192696400 |
| 29887018 | G | A | 3' UTR | Modifier | 4 | - | c.*326G>A |  | - |
| 29887053 | G | C | 3' UTR | Modifier | 4 | - | c.*361G>C |  | rs185039421 |
| 29887072 | C | T | 3' UTR | Modifier | 4 | - | c.*380C>T |  | rs978569780 |
| 29887112 | TTC | - | 3' UTR | Modifier | 4 | - | c.*420_*421del |  | rs1490643558 |
| 29887148 | G | A | 3' UTR | Modifier | 4 | - | c.*456G>A |  | - |
| 29887151 | C | G | 3' UTR | Modifier | 4 | - | c.*459C>G |  | rs540106485 |
| 29887182 | C | T | 3' UTR | Modifier | 4 | - | c.*490C>T |  | rs142042497 |
| 29887186 | G | C | 3' UTR | Modifier | 4 | - | c.*494G>C |  | rs710195 |
| 29887211 | C | T | 3' UTR | Modifier | 4 | - | c.*519C>T |  | rs911785075 |
| 29887212 | G | A | 3' UTR | Modifier | 4 | - | c.*520G>A |  | rs944633264 |
| 29887216 | T | C | 3' UTR | Modifier | 4 | - | c.*524T>C |  | rs1218993302 |
| 29887227 | G | T | 3' UTR | Modifier | 4 | - | c.*535G>T |  | rs772755733 |
| 29887238 | A | G | 3' UTR | Modifier | 4 | - | c.*546A>G |  | rs561850196 |
| 29887249 | G | A | 3' UTR | Modifier | 4 | - | c.*557G>A |  | rs1321602381 |
| 29887271 | A | T | 3' UTR | Modifier | 4 | - | c.*579A>T |  | - |
| 29887315 | G | C | 3' UTR | Modifier | 4 | - | c.*623G>C |  | - |

Supplementary Table 7. Details from Ensembl Variant Effect Predictor (VEP) of all the 591 variants (hg19) identified in the Project MinE dataset. HGVS.c notation refers to the ENST00000310624.6 transcript. HGVS.p notation refers to the ENSP00000311997.6 protein transcript.

| **Variant** | **Position** | **Type** | **Pathogenicity Prediction** | **CADD-SV Score** | **Total Carriers (Case/Control)** | **Beta (SE)** | **OR**  **(95% CI)** | **P-Value** |
| --- | --- | --- | --- | --- | --- | --- | --- | --- |
| A90V | 29876520 | Missense | Tolerated/Benign | N/A | 7 (6/1) | 0.49 (0.95) | 2.26 (0.27-18.77) | 0.61 |
| G249S | 29876996 | Missense | Tolerated/Benign | N/A | 92 (69/23) | 0.03 (0.23) | 1.13 (0.70-1.82) | 0.91 |
| A314V | 29879421 | Missense | Deleterious/ Probably Damaging | N/A | 1 (1/0) | 0.22 (2.11) | 1.13 (0.05-27.73) | 0.92 |
| E463K | 29885016 | Missense | Deleterious/ Probably Damaging | N/A | 1527 (1103/424) | -0.0065 (0.06) | 0.97 (0.86-1.10) | 0.91 |
| T642M | 29885554 | Missense | Tolerated/Benign | N/A | 1 (0/1) | -2.47 (2.11) | 0.13 (0.005-3.08) | 0.24 |
| K647N | 29885570 | Missense | Deleterious/ Possibly Damaging | N/A | 1 (1/0) | 0.40 (2.12) | 1.13 (0.05-27.73) | 0.85 |
| P777L | 29885959 | Missense | Deleterious/ Probably Damaging | N/A | 1 (1/0) | 0.14 (2.12) | 1.13 (0.05-27.73) | 0.95 |
| E805A | 29886043 | Missense | Deleterious/ Possibly Damaging | N/A | 2578 (1873/705) | -0.028 (0.05) | 1.00 (0.90-1.11) | 0.54 |
| K867N | 29886230 | Missense | Deleterious/ Probably Damaging | N/A | 1 (1/0) | 0.40 (2.12) | 1.13 (0.05-27.73) | 0.85 |
| A400A | 29881828 | Synonymous | N/A | N/A | 2569 (1867/702) | 0.03 (0.05) | 1.00 (0.90-1.11) | 0.57 |
| S580S | 29885369 | Synonymous | N/A | N/A | 199 (147/52) | 0.10 (0.16) | 1.07 (0.77-1.47) | 0.56 |
| D919D | 29886386 | Synonymous | N/A | N/A | 148 (105/43) | -0.05 (0.18) | 0.92 (0.64-1.31) | 0.77 |
| V928V | 29886413 | Synonymous | N/A | N/A | 8492 (6198/2324) | -0.007 (0.04) | 1.08 (0.86-1.36) | 0.85 |
| K790del | 29885996 | Small Deletion | N/A | N/A | 18 (9/9) | -1.03 (0.47) | 0.38 (0.15-0.95) | **0.03** |
| E658_K665del | 29885604 | Small Deletion | N/A | N/A | 29 (18/11) | 0.02 (0.64) | 0.61 (0.29-1.30) | 0.98 |
| E664_P669del | 29885622 | Small Deletion | N/A | N/A | 13 (10/3) | -2.20 (2.11) | 1.25 (0.35-4.56) | 0.3 |
| INS_61 | 29877834-29877895 | Large Deletion | N/A | 14.27 | 1 (1/0) | 0.47 (2.11) | 1.19 (0.049- 29.24) | 0.82 |
| INS_56 | 29879816-29879872 | Large Deletion | N/A | 0.75 | 1 (0/1) | -2.38 (2.11) | 0.13 (0.0054- 3.25) | 0.26 |
| DEL_1169 | 29880841-29882010 | Large Deletion | Likely Pathogenic | 9.83 | 1 (1/0) | 0.65 (2.11) | 1.19 (0.049- 29.24) | 0.76 |
| DEL_113 | 29885279-29885870 | Large Deletion | VUS | 4.85 | 1743 (1161/582) | -0.34 (0.061) | 0.72 (0.64-0.81) | **2.60E-08** |

Supplementary Table 8. Case-control frequencies and results of logistic regression association analysis of the 16 SNV/indel variants found in both the systematic review and Project MinE, and the 4 structural variants identified in a subset of Project MinE. Results were corrected for sex and 10 principal components. N/A refers to a pathogenicity prediction that could not be reached as the variant type was inappropriate for the tool (SIFT/PolyPhen for SNVs and indels; ACMG and CADD-SV for SVs). VUS = variant of unknown significance.

| **Variant** | **No. Studies** | **N Cases** | **N Controls** | **Fixed OR**  **(95% CI; p-value)** | **Random OR**  **(95% CI; p-value)** | **Heterogeneity**  **(Q statistic; p-value)** | **Heterogeneity**  **(I-squared)** | **Publication Bias (Egger t statistic; p-value)** | **Publication Bias (Harbord t statistic; p-value)** |
| --- | --- | --- | --- | --- | --- | --- | --- | --- | --- |
| A90V | 2 | 6569 | 2534 | 2.45  (0.42-14.51; 0.32) | 2.47  (0.42-14.47; 0.32) | 0.02 (0.88) | 0% | N/A | N/A |
| G249S | 3 | 6940 | 3245 | 1.10  (0.71-1.70; 0.68) | 1.10  (0.70-1.70; 0.69) | 0.79 (0.67) | 0% | 0.14; 0.91 | 0.40; 0.76 |
| A314V | 2 | 6840 | 3145 | 2.61  (0.27-25.00; 0.41) | 2.55  (0.26-24.53; 0.42) | 0.50 (0.48) | 0% | N/A | N/A |
| E463K | 2 | 7595 | 3047 | 0.95  (0.85-1.06; 0.36) | 0.95  (0.85-1.06; 0.36) | 1.01 (0.31) | 2% | N/A | N/A |
| T642M | 4 | 7197 | 4044 | 1.39  (0.53-3.16; 0.56) | 1.23  (0.46-3.28; 0.69) | 3.29 (0.35) | 9% | -0.44; 0.71 | -1.21; 0.35 |
| E658_K665del | 3 | 7047 | 3364 | 0.68  (0.35-1.32; 0.25) | 0.66  (0.34-1.29; 0.23) | 0.96 (0.62) | 0% | 1.59; 0.36 | 2.58; 0.24 |
| E664_P669del | 2 | 6676 | 2653 | 1.22  (0.38-3.92; 0.74) | 1.22  (0.38-3.93; 0.74) | 0.01 (0.91) | 0% | N/A | N/A |
| K790del | 3 | 7951 | 3353 | 0.60  (0.28-1.29; 0.19) | 0.63  (0.13-3.01; 0.57) | 3.97 (0.14) | **50%** | 0.81; 0.57 | 0.55; 0.68 |
| E805A | 3 | 7966 | 3758 | 1.01  (0.93-1.11; 0.76) | 1.02  (0.93-1.11; 0.75) | 2.89 (0.24) | 31% | N/A | N/A |
| K867N | 2 | 6651 | 2624 | 1.94  (0.20-18.60; 0.57) | 1.88  (0.20-18.15; 0.58) | 0.20 (0.66) | 0% | N/A | N/A |

Supplementary Table 9. Results of variant-level meta-analysis when including the Project MinE cohort as an additional study. Meta-analysis was repeated for 5 variants (G249S, T642M, E658_K665del, K790del, E805A)), and new meta-analysis was performed for 5 variants (A90V, A314V, E463K, E664_P669del, K867N), who now have sufficient evidence to be included (≥2 case-control studies). N/A values represent instances where publication bias could not be calculated as the minimum number of studies used for calculation was not reached.

| **Frequency (<0.1%)** | **N. of variants** | **Madsen Browning p** | **SKAT-O p** | **Cases** | **Controls** | **Odds Ratio (95% CI)** |
| --- | --- | --- | --- | --- | --- | --- |
| *All (29876207-29887379)* | 512 | **7.0E-04** | **3.3E-03** | 595 | 168 | 1.37 (1.14-1.63) |
| Synonymous | 37 | 0.12 | 0.43 | 47 | 12 | 1.48 (0.78-2.79) |
| Missense | 80 | 0.11 | 0.25 | 85 | 24 | 1.34 (0.85-2.11) |
| All pathogenic missense | 51 | 0.11 | 0.37 | 51 | 13 | 1.48 (0.80-2.73) |
| Inframe Deletion | 11 | 0.051 | 0.35 | 16 | 2 | 3.01 (0.69-13.12) |
| Inframe Insertion | 11 | 0.38 | 0.75 | 12 | 3 | 1.51 (0.42-5.34) |
| Intronic | 332 | **4.3E-03** | **0.01** | 419 | 115 | 1.40 (1.13-1.73) |
| 3’ UTR | 30 | 0.29 | 0.72 | 39 | 12 | 1.22 (0.64-2.34) |
| 5’ UTR | 4 | **4.0E-03** | **0.037** | 1 | 3 | 0.13 (0.01-1.21) |
| VEP High | 6 | 0.13 | 0.11 | 3 | 4 | 0.28 (0.06-1.26) |
| VEP Low | 37 | 0.12 | 0.43 | 47 | 12 | 1.48 (0.78-2.79) |
| VEP Moderate | 102 | **0.032** | 0.16 | 113 | 29 | 1.47 (0.98-2.22) |
| VEP Modifier | 367 | **4.5E-03** | **0.012** | 458 | 130 | 1.35 (1.10-1.65) |
| *Head (29876252-29876554)* | 13 | 0.76 | 0.26 | 15 | 9 | 0.63 (0.27-1.43) |
| Synonymous | 7 | 0.49 | 0.24 | 8 | 3 | 1.00 (0.27-3.79) |
| Missense | 6 | 0.091 | 0.17 | 7 | 6 | 0.44 (0.15-1.31) |
| Pathogenic missense | 1 | N/A | 0.069 | 1 | 0 | 1.13 (0.05-27.73) |
| VEP Low | 7 | 0.49 | 0.24 | 8 | 3 | 1.00 (0.27-3.79) |
| VEP Moderate | 6 | 0.091 | 0.17 | 7 | 6 | 0.44 (0.15-1.31) |
| *Rod (29876555-29884868)* | 379 | **7.0E-04** | **3.0E-03** | 473 | 126 | 1.45 (1.18-1.77) |
| Synonymous | 20 | 0.08 | 0.25 | 28 | 5 | 2.11 (0.81-5.48) |
| Missense | 23 | 0.065 | 0.25 | 29 | 6 | 1.82 (0.76-4.39) |
| Pathogenic missense | 14 | 0.48 | 0.73 | 15 | 5 | 1.13 (0.41-3.11) |
| Inframe Insertion | 1 | 0.26 | 0.53 | 1 | 0 | 1.13 (0.041-27.73) |
| Intronic | 332 | **4.0E-03** | **0.01** | 419 | 115 | 1.40 (1.13-1.73) |
| VEP High | 3 | 0.18 | 0.20 | 1 | 2 | 0.19 (0.02-2.07) |
| VEP Low | 20 | 0.08 | 0.25 | 28 | 5 | 2.11 (0.81-5.48) |
| VEP Moderate | 24 | 0.052 | 0.22 | 30 | 6 | 1.89 (0.78-4.54) |
| VEP Modifier | 332 | **0.004** | **0.01** | 419 | 115 | 1.40 (1.13-1.73) |
| *Tail (29884869-29886689)* | 85 | **0.057** | 0.30 | 89 | 23 | 1.46 (0.92-2.32) |
| Synonymous | 10 | 0.65 | 0.71 | 11 | 4 | 1.03 (0.33-3.25) |
| Missense | 51 | 0.084 | 0.21 | 49 | 12 | 1.54 (0.82-2.90) |
| Pathogenic Missense | 36 | **0.039** | 0.19 | 36 | 7 | 1.94 (0.86-4.37) |
| E Segment | 8 | 0.48 | 0.68 | 6 | 2 | 1.19 (0.23-5.60) |
| KSP Segment | 17 | 0.34 | 0.73 | 16 | 4 | 1.51 (0.50-4.51) |
| KEP Segment | 11 | **0.02** | 0.11 | 15 | 1 | 5.65 (0.75-42.83) |
| Inframe Deletion | 11 | 0.052 | 0.35 | 16 | 2 | 3.01 (0.69-13.12) |
| Inframe Insertion | 10 | 0.43 | 0.84 | 11 | 3 | 1.38 (0.38-4.95) |
| VEP High | 3 | 0.36 | 0.35 | 2 | 2 | 0.38 (0.05-2.67) |
| VEP Low | 10 | 0.65 | 0.71 | 11 | 4 | 1.03 (0.33-3.25) |
| VEP Moderate | 72 | **0.024** | 0.15 | 76 | 17 | 1.69 (1.00-2.87) |
| *All excluding tail* | 392 | **3.0E-03** | **7.5E-03** | 478 | 133 | 1.38 (1.13-1.68) |

Supplementary Table 10. Results of the ultra-rare (MAF < 0.1%) NEFH variant burden analysis. Significant results are denoted by bold p-values. All results are corrected for sex and the first 10 principal components.

| **Frequency (0.1-1%)** | **N. of variants** | **Madsen Browning p** | **SKAT-O p** | **Cases** | **Controls** | **Odds Ratio (95% CI)** |
| --- | --- | --- | --- | --- | --- | --- |
| *All (29876207-29887379)* | 45 | 0.78 | 0.61 | 735 | 285 | 0.97 (0.84-1.12) |
| Synonymous | 4 | 0.97 | 1.00 | 38 | 13 | 1.10 (0.59-2.07) |
| Missense | 7 | 0.26 | 0.58 | 91 | 34 | 1.01 (0.68-1.50) |
| All pathogenic missense | 5 | 0.27 | 0.39 | 18 | 9 | 0.75 (0.34-1.68) |
| Inframe Deletion | 3 | 0.44 | **0.03** | 50 | 16 | 1.18 (0.67-2.07) |
| Intronic | 29 | 0.82 | 0.64 | 625 | 240 | 0.98 (0.84-1.14) |
| 3’ UTR | 2 | 0.82 | 0.80 | 21 | 9 | 0.88 (0.40-1.92) |
| VEP Low | 4 | 0.97 | 1.00 | 38 | 13 | 1.10 (0.59-2.07) |
| VEP Moderate | 10 | 0.69 | 0.27 | 141 | 50 | 1.06 (0.77-1.47) |
| VEP Modifier | 31 | 0.82 | 0.64 | 644 | 248 | 0.97 (0.84-1.14) |
| *Head (29876252-29876554)* | - | - | - | - | - | - |
| *Rod (29876555-29884868)* | 34 | 0.60 | 0.56 | 641 | 252 | 0.95 (0.82-1.11) |
| Missense | 5 | 0.11 | 0.52 | 81 | 32 | 0.95 (0.63-1.44) |
| Intronic | 29 | 0.82 | 0.64 | 625 | 240 | 0.98 (0.84-1.14) |
| VEP Moderate | 5 | 0.11 | 0.52 | 81 | 32 | 0.95 (0.63-1.44) |
| VEP Modifier | 29 | 0.82 | 0.64 | 625 | 240 | 0.98 (0.84-1.14) |
| *Tail (29884869-29886689)* | 9 | 0.71 | 0.22 | 95 | 33 | 1.08 (0.73-1.62) |
| Synonymous | 4 | 0.96 | 1.00 | 38 | 13 | 1.10 (0.59-2.07) |
| Missense | 2 | 0.91 | 0.73 | 16 | 6 | 1.00 (0.39-2.57) |
| Inframe Deletion | 3 | 0.45 | **0.03** | 50 | 16 | 1.18 (0.67-2.07) |
| VEP Low | 4 | 0.96 | 1.00 | 38 | 13 | 1.10 (0.59-2.07) |
| VEP Moderate | 5 | 0.58 | **0.04** | 66 | 22 | 1.13 (0.70-1.84) |
| *All excluding tail* | 34 | 0.60 | 0.56 | 641 | 252 | 0.95 (0.82-1.11) |

Supplementary Table 11. Results of the high-frequency rare (MAF 0.1-1%) NEFH variant burden analysis. Significant results are denoted by bold p-values. All results are corrected for sex and the first 10 principal components.

| **Frequency (<1%)** | **N. of variants** | **Madsen Browning p** | **SKAT-O p** | **Cases** | **Controls** | **Odds Ratio (95% CI)** |
| --- | --- | --- | --- | --- | --- | --- |
| *All (29876207-29887379)* | 557 | **0.03** | 0.38 | 1269 | 438 | 1.11 (0.99-1.25) |
| Synonymous | 41 | 0.11 | 0.32 | 47 | 12 | 1.48 (0.78-2.79) |
| Missense | 87 | 0.11 | 0.56 | 85 | 24 | 1.34 (0.85-2.11) |
| All pathogenic missense | 56 | 0.36 | 0.8 | 69 | 22 | 1.18 (0.73-1.91) |
| Inframe Deletion | 15 | **0.05** | 0.42 | 16 | 2 | 3.01 (0.69-13.12) |
| Inframe Insertion | 11 | 0.38 | 0.75 | 12 | 3 | 1.51 (0.42-5.34) |
| Intronic | 361 | **4.0E-03** | **0.034** | 419 | 115 | 1.40 (1.13-1.73) |
| 3’ UTR | 31 | 0.22 | 0.66 | 39 | 11 | 1.34 (0.68-2.61) |
| 5’ UTR | 4 | **4.0E-03** | **0.037** | 1 | 3 | 0.13 (0.01-1.21) |
| VEP High | 6 | 0.13 | 0.11 | 3 | 4 | 0.28 (0.06-1.26) |
| VEP Low | 41 | 0.11 | 0.32 | 47 | 12 | 1.48 (0.78-2.79) |
| VEP Moderate | 113 | **0.029** | 0.18 | 113 | 29 | 1.47 (0.98-2.22) |
| VEP Modifier | 397 | **3.1E-03** | **0.034** | 460 | 127 | 1.39 (1.14-1.70) |
| *Head (29876252-29876554)* | 13 | 0.76 | 0.26 | 15 | 9 | 0.63 (0.27-1.43) |
| Synonymous | 7 | 0.49 | 0.24 | 8 | 3 | 1.00 (0.27-3.79) |
| Missense | 6 | 0.091 | 0.17 | 7 | 6 | 0.44 (0.15-1.31) |
| Pathogenic missense | 1 | N/A | 0.069 | 1 | 0 | 1.13 (0.05-27.73) |
| VEP Low | 7 | 0.49 | 0.24 | 8 | 3 | 1.00 (0.27-3.79) |
| VEP Moderate | 6 | 0.091 | 0.17 | 7 | 6 | 0.44 (0.15-1.31) |
| *Rod (29876555-29884868)* | 413 | **0.03** | 0.32 | 1062 | 363 | 1.12 (0.98-1.28) |
| Synonymous | 20 | 0.08 | 0.25 | 28 | 5 | 2.11 (0.81-5.48) |
| Missense | 28 | 0.07 | 0.55 | 29 | 6 | 1.82 (0.76-4.39) |
| Pathogenic missense | 17 | 0.85 | 0.46 | 23 | 12 | 0.72 (0.36-1.45) |
| Inframe Insertion | 1 | 0.26 | 0.53 | 1 | 0 | 1.13 (0.041-27.73) |
| Intronic | 361 | **4.0E-03** | **0.034** | 419 | 115 | 1.40 (1.13-1.73) |
| VEP High | 3 | 0.18 | 0.2 | 1 | 2 | 0.19 (0.02-2.07) |
| VEP Low | 20 | 0.08 | 0.25 | 28 | 5 | 2.11 (0.81-5.48) |
| VEP Moderate | 29 | **0.05** | 0.48 | 30 | 6 | 1.89 (0.78-4.54) |
| VEP Modifier | 397 | **3.1E-03** | **0.034** | 460 | 127 | 1.39 (1.14-1.70) |
| *Tail (29884869-29886689)* | 95 | 0.18 | 0.21 | 193 | 63 | 1.15 (0.86-1.54) |
| Synonymous | 14 | 0.65 | 0.89 | 10 | 4 | 0.94 (0.29-3.00) |
| Missense | 53 | 0.08 | 0.41 | 49 | 12 | 1.54 (0.82-2.90) |
| Pathogenic Missense | 38 | 0.09 | 0.36 | 52 | 13 | 1.51 (0.82-2.78) |
| Inframe Deletion | 15 | **0.05** | 0.42 | 16 | 2 | 3.01 (0.69-13.12) |
| Inframe Insertion | 10 | 0.43 | 0.84 | 11 | 3 | 1.38 (0.38-4.95) |
| VEP High | 3 | 0.36 | 0.35 | 2 | 2 | 0.38 (0.05-2.67) |
| VEP Low | 14 | 0.65 | 0.89 | 10 | 4 | 0.94 (0.29-3.00) |
| VEP Moderate | 78 | **0.024** | 0.28 | 76 | 17 | 1.69 (1.00-2.87) |
| *All excluding tail* | 431 | 0.06 | 0.43 | 1068 | 372 | 1.10 (0.96-1.25) |

Supplementary Table 12. Results of the rare (MAF < 1%) NEFH variant burden analysis. Significant results are denoted by bold p-values. All results are corrected for sex and the first 10 principal components.

| **Variant** | **Start** | **End** | **Ref** | **Alt** | **SIFT** | **PolyPhen** | **REVEL** | **CADD** | **Total Frequency (Case/Control)** |
| --- | --- | --- | --- | --- | --- | --- | --- | --- | --- |
| G414S | 29884869 | 29884869 | G | A | Tolerated | Possibly Damaging | 0.296 | 24.0 | 1 (0/1) |
| K437R | 29884939 | 29884939 | A | G | Deleterious | Probably Damaging | 0.403 | 25.1 | 1 (1/0) |
| K437N | 29884940 | 29884940 | G | T | Deleterious | Probably Damaging | 0.533 | 23.0 | 1 (1/0) |
| E441K | 29884950 | 29884950 | G | A | Deleterious | Probably Damaging | 0.368 | 28.1 | 1 (0/1) |
| K449N | 29884976 | 29884976 | G | C | Deleterious | Probably Damaging | 0.382 | 23.4 | 1 (1/0) |
| E477Q | 29885058 | 29885058 | G | C | Deleterious | Possibly Damaging | 0.455 | 25.5 | 1 (1/0) |
| P512R | 29885164 | 29885164 | C | G | Deleterious | Possibly Damaging | 0.396 | 24.9 | 1 (1/0) |
| E523D | 29885198 | 29885198 | G | C | Deleterious | Benign | 0.228 | 8.57 | 1 (1/0) |
| K525E | 29885202 | 29885202 | A | G | Deleterious | Benign | 0.187 | 19.2 | 1 (1/0) |
| S560L | 29885308 | 29885308 | C | T | Deleterious | Benign | 0.276 | 14.2 | 1 (1/0) |
| P561L | 29885311 | 29885311 | C | T | Deleterious | Benign | 0.271 | 15.8 | 1 (1/0) |
| K585R | 29885383 | 29885383 | A | G | Deleterious | Possibly Damaging | 0.382 | 22.7 | 2 (1/1) |
| S586F | 29885386 | 29885386 | C | T | Deleterious | Benign | 0.132 | 18.8 | 1 (0/1) |
| A592S | 29885403 | 29885403 | G | T | Deleterious | Benign | 0.061 | 15.6 | 2 (2/0) |
| K599T | 29885425 | 29885425 | A | C | Deleterious | Probably Damaging | 0.447 | 23.2 | 1 (0/1) |
| K627Q | 29885508 | 29885508 | A | C | Deleterious | Benign | 0.252 | 19.9 | 1 (1/0) |
| K647N | 29885570 | 29885570 | G | C | Deleterious (Low Confidence) | Possibly Damaging | 0.365 | 14.7 | 1 (1/0) |
| E658_E659del | 29885598 | 29885598 | AAGGAAG | A | - | - | - | 11.8 | 36 (31/5) |
| E664_P669del | 29885604 | 29885604 | GAGGCCAAGTCCCCTGAGA | G | - | - | - | 14.1 | 13 (10/3) |
| K671Q | 29885640 | 29885640 | A | C | Deleterious (Low Confidence) | Possibly Damaging | 0.257 | 18.43 | 2 (1/1) |
| K703Q | 29885736 | 29885736 | A | C | Deleterious (Low Confidence) | Probably Damaging | 0.345 | 22.2 | 1 (1/0) |
| P705S | 29885742 | 29885742 | C | T | Deleterious (Low Confidence) | Probably Damaging | 0.372 | 20.6 | 1 (1/0) |
| A736S | 29885835 | 29885835 | G | T | Deleterious (Low Confidence) | Benign | 0.338 | 22.5 | 1 (1/0) |
| S752F | 29885884 | 29885884 | C | T | Deleterious (Low Confidence) | Possibly Damaging | 0.333 | 24.7 | 1 (1/0) |
| P777L | 29885959 | 29885959 | C | T | Deleterious (Low Confidence) | Probably Damaging | 0.476 | 24.1 | 1 (1/0) |
| K790del | 29885996 | 29885996 | CAAG | C | - | - | - | 10.6 | 18 (9/9) |
| E791A | 29886001 | 29886001 | A | C | Deleterious (Low Confidence) | Benign | 0.266 | 22.0 | 1 (1/0) |
| P802L | 29886034 | 29886034 | C | T | Deleterious (Low Confidence) | Probably Damaging | 0.221 | 16.9 | 1 (1/0) |
| K840E | 29886147 | 29886147 | A | G | Tolerated (Low Confidence) | Possibly Damaging | 0.443 | 23.7 | 1 (1/0) |
| K867N | 29886230 | 29886230 | G | T | Deleterious (Low Confidence) | Probably Damaging | 0.427 | 21.2 | 1 (1/0) |
| P880L | 29886268 | 29886268 | C | T | Deleterious (Low Confidence) | Benign | 0.226 | 18.7 | 1 (1/0) |
| E883K | 29886276 | 29886276 | G | A | Deleterious (Low Confidence) | Probably Damaging | 0.422 | 24.8 | 1 (1/0) |
| E887G | 29886289 | 29886289 | A | G | Deleterious (Low Confidence) | Benign | 0.344 | 23.2 | 1 (1/0) |
| K908R | 29886352 | 29886352 | A | G | Deleterious (Low Confidence) | Benign | 0.294 | 21.9 | 1 (1/0) |
| V916M | 29886375 | 29886375 | G | A | Deleterious (Low Confidence) | Benign | 0.242 | 13.2 | 4 (4/0) |
| E924A | 29886400 | 29886400 | A | C | Deleterious (Low Confidence) | Benign | 0.221 | 22.0 | 2 (2/0) |
| A929D | 29886415 | 29886415 | C | A | Deleterious (Low Confidence) | Benign | 0.228 | 19.66 | 1 (0/1) |
| A950V | 29886478 | 29886478 | C | T | Deleterious (Low Confidence) | Probably Damaging | 0.283 | 22.0 | 2 (2/0) |
| K1015R | 29886673 | 29886673 | A | G | Deleterious (Low Confidence) | Benign | 0.35 | 22.5 | 1 (1/0) |

Supplementary Table 13. Genomic coordinates (hg19) and pathogenicity prediction of the ultra-rare pathogenic missense and high-frequency rare in-frame deletion variants which were found to significantly increase the risk of ALS when performing burden analysis in the Project MinE dataset.

| Variant Class | Method | No. Cases | Male:Female (Ratio) | Age of Onset in Years (Mean ± SD) | Diagnostic Delay in Years (Mean ± SD) | Age at Death in Years (Mean ± SD) | Disease Duration in Years (Median (IQR)) | Male:Female (Ratio) (X2,p-value) | Age of Onset in Years (Mean ± SD; F statistic, p-value) | Diagnostic Delay in Years (Mean ± SD; F statistic, p-value) | Disease Duration in Years (Median (IQR); Hazard Ratio (95% CI), p-value) |
| --- | --- | --- | --- | --- | --- | --- | --- | --- | --- | --- | --- |
| K790del | Firth Regression | 9 | 6:3 (2.00) | 67.42 ± 7.63 | 0.91 ± 0.77 | 70.96 ± 6.24 | 3.06 (1.83) | 0.181, 0.671 | 7.92 ± 4.17; 1.898, 0.0578 | -0.36 ± 0.51; 0.490, 0.484 | 0.43 (0.41); 1.22 (0.58-2.56),0.599 |
| 113bp deletion | Firth Regression | 1161 | 697:464 (1.50) | 62.69 ± 11.54 | 1.18 ± 1.49 | 66.87 ± 10.21 | 2.46 (2.08) | 0.059, 0.808 | 2.87 ± 0.42; 46.802, **8.66E-12** | -0.07 ± 0.05; 1.666, 0.197 | -0.20 (0.16); 1.10 (1.03-1.19), **7.00E-03** |
| Whole Gene (< 0.1%) | Burden Analysis | 595 | 338:257 (1.32) | 59.33 ± 12.85 | 1.28 ± 1.37 | 65.51 ± 11.03 | 2.67 (2.26) | 2.310, 0.129 | -0.97 ± 0.57; 2.954, 0.0858 | 0.08 ± 0.07; 1.099, 0.295 | 0.055 (0.018); 0.95 (0.85-1.06), 0.353 |
| Whole Gene exc. Tail (< 0.1%) | Burden Analysis | 478 | 277:201 (1.38) | 59.25 ± 12.90 | 1.23 ± 1.33 | 65.49 ± 10.89 | 2.62 (2.33) | 0.671, 0.413 | -1.03 ± 0.62; 2.741, 0.0978 | 0.01 ± 0.08; 0.023, 0.880 | 0.0027 (0.10); 0.97 (0.86-1.09), 0.580 |
| Rod (< 0.1%) | Burden Analysis | 473 | 273:200 (1.37) | 59.20 ± 12.95 | 1.23 ± 1.33 | 65.50 ± 10.93 | 2.62 (2.34) | 0.850, 0.357 | -1.11 ± 0.63; 3.103, 0.0782 | 0.02 ± 0.08; 0.038, 0.845 | 0.0027 (0.10); 0.96 (0.85-1.08), 0.511 |
| Tail (< 0.1%) | Burden Analysis | 89 | 41:48 (0.85) | 58.55 ± 12.41 | 1.48 ± 1.66 | 64.96 ± 11.61 | 2.98 (2.06) | 6.991, **8.20E-03** | -1.78 ± 1.42; 1.574, 0.210 | 0.35 ± 0.18; 3.698, 0.0545 | 0.36 (0.18); 0.80 (0.60-1.06), 0.118 |
| Tail Pathogenic Missense (< 0.1%) | Burden Analysis | 36 | 15:21 (0.71) | 59.24 ± 10.99 | 1.72 ± 2.13 | 66.82 ± 9.93 | 3.08 (2.27) | 4.904, **0.0268** | -1.34 ± 2.15; 0.387, 0.534 | 0.55 ± 0.28; 4.021, **0.0450** | 0.46 (0.023); 0.80 (0.52-1.22), 0.298 |
| Tail (KEP repeat) Pathogenic Missense (< 0.1%) | Burden Analysis | 15 | 8:7 (1.14) | 62.46 ± 7.08 | 2.03 ± 2.76 | 67.41 ± 8.44 | 3.67 (3.52) | 0.255, 0.614 | 2.37 ± 3.35; 0.501, 0.479 | 0.91 ± 0.44; 4.314, **0.0379** | 1.05 (1.27); 0.77 (0.38-1.54), 0.456 |
| Tail In-Frame Deletions (0.1-1%) | Burden Analysis | 50 | 32:18 (1.78) | 61.91 ± 12.20 | 1.84 ± 2.63 | 66.14 ± 10.99 | 2.43 (1.41) | 0.384, 0.536 | 1.58 ± 1.78; 0.792, 0.374 | 0.63 ± 0.22; 8.404, **3.76E-03** | -0.19 (0.84); 1.12 (0.82-1.54), 0.470 |
| Tail VEP Moderate (0.1-1%) | Burden Analysis | 66 | 43:23 (1.87) | 61.51 ± 12.05 | 1.60 ± 2.37 | 65.31 ± 11.35 | 2.42 (1.50) | 0.818, 0.366 | 1.29 ± 1.56; 0.685, 0.408 | 0.42 ± 0.19; 4.705, **0.0301** | -0.20 (0.74); 1.15 (0.87-1.52), 0.322 |
| Whole Gene (< 1%) | Burden Analysis | 1269 | 745:524 (1.42) | 59.86 ± 12.77 | 1.33 ± 1.72 | 65.62 ± 11.01 | 2.61 (2.19) | 0.671, 0.413 | -0.27 ± 0.41; 0.443, 0.506 | 0.11 ± 0.05; 4.699, **0.0302** | -0.018 (0.056); 1.05 (0.97-1.13), 0.251 |
| Whole Gene VEP Moderate (< 1%) | Burden Analysis | 113 | 55:58 (0.95) | 58.70 ± 11.93 | 1.46 ± 1.58 | 65.62 ± 11.19 | 3.08 (2.99) | 5.833, **0.0157** | -1.75 ± 1.26; 1.919, 0.166 | 0.32 ± 0.16; 4.026, **0.0449** | 0.47 (0.75); 0.74 (0.58-0.94), **0.0147** |
| Rod (< 1%) | Burden Analysis | 1062 | 625:437 (1.43) | 59.71 ± 12.95 | 1.27 ± 1.71 | 65.51 ± 11.15 | 2.57 (2.25) | 0.397, 0.529 | -0.45 ± 0.44; 1.037, 0.309 | 0.036 ± 0.057; 0.399, 0.528 | -0.058 (0.012); 1.06 (0.98-1.16), 0.145 |
| Rod Intronic (<1%) | Burden Analysis | 419 | 239:180 (1.33) | 59.48 ± 13.05 | 1.22 ± 1.34 | 65.49 ± 11.21 | 2.58 (2.35) | 1.336, 0.248 | -0.81 ± 0.66; 1.477, 0.224 | 0.0026 ± 0.086; 0.000900, 0.976 | -0.038 (0.12); 0.98 (0.87-1.11), 0.778 |
| Rod VEP Moderate (< 1%) | Burden Analysis | 30 | 17:13 (1.31) | 58.55 ± 11.22 | 1.74 ± 1.50 | 68.27 ± 6.68 | 3.77 (6.58) | 0.117, 0.733 | -1.72 ± 2.46; 0.491, 0.484 | 0.59 ± 0.31; 3.590, 0.0582 | 1.16 (4.34); 0.62 (0.38-1.02), 0.0585 |
| Rod VEP Modifier (< 1%) | Burden Analysis | 460 | 264:196 (1.35) | 59.65 ± 12.97 | 1.24 ± 1.31 | 65.48 ± 11.22 | 2.62 (2.27) | 1.115, 0.291 | -0.63 ± 0.64; 0.990, 0.320 | 0.014 ± 0.082; 0.0306, 0.861 | -0.0041 (0.027); 0.99 (0.88-1.12), 0.873 |
| Tail In-Frame Deletions (< 1%) | Burden Analysis | 16 | 7:9 (0.78) | 59.16 ± 11.47 | 1.22 ± 0.81 | 66.06 ± 11.45 | 3.46 (3.13) | 1.700, 0.192 | -2.38 ± 3.23; 0.542, 0.462 | 0.038 ± 0.39; 0.00960, 0.922 | 0.85 (0.88); 0.67 (0.37-1.21), 0.181 |
| Tail VEP Moderate (< 1%) | Burden Analysis | 76 | 35:41 (0.85) | 58.81 ± 12.05 | 1.43 ± 1.68 | 64.87 ± 11.97 | 3.14 (2.06) | 5.970, **0.0145** | -1.56 ± 1.53; 1.049, 0.306 | 0.30 ± 0.19; 2.439, 0.118 | 0.53 (0.18); 0.80 (0.60-1.07), 0.126 |
| Individuals with no variant classes | NA | 4277 | 2560:1717 (1.49) | 59.56 ± 12.85 | 1.26 ± 1.44 | 65.14 ± 11.08 | 2.67 (2.26) | 0.098, 0.754 | -1.28 ± 0.34; 13.739, **2.12E-04** | -0.015 ± 0.043; 0.129, 0.719 | 0.13 (0.078); 0.92 (0.87-0.98), **9.40E-03** |

Supplementary Table 14: Demographics of people with ALS in variant classes significantly associated with ALS risk from Firth regression and rare variant burden analysis (columns D-H), with statistical comparison of male:female ratio, age of onset, diagnostic delay and disease duration between those in each variant class versus those absent from each variant class (columns I-L). Differences in male:female ratio was assessed with the Chi-squared test. Differences in age of onset and diagnostic delay was assessed using 2-way ANOVA corrected for sex and site of onset, with differences in disease duration assessed using a Cox proportional hazards model corrected for sex and site of onset. Nominally significant results are denoted by bold p-values. IQR stands for interquartile range.

REFERENCES

1. Figlewicz DA, Krizus A, Martinoli MG, Meininger V, Dib M, Rouleau GA, et al. Variants of the heavy neurofilament subunit are associated with the development of amyotrophic lateral sclerosis. Hum Mol Genet. 1994 Oct;3(10):1757–61.

2. Vechio JD, Bruijn LI, Xu Z, Brown RH, Cleveland DW. Sequence variants in human neurofilament proteins: absence of linkage to familial amyotrophic lateral sclerosis. Ann Neurol. 1996 Oct;40(4):603–10.

3. Tomkins J, Usher P, Slade JY, Ince PG, Curtis A, Bushby K, et al. Novel insertion in the KSP region of the neurofilament heavy gene in amyotrophic lateral sclerosis (ALS). Neuroreport. 1998 Dec 1;9(17):3967–70.

4. Al-Chalabi A, Andersen P, Nilsson P, Chioza B, Andersson J, Russ C, et al. Deletions of the heavy neurofilament subunit tail in amyotrophic lateral sclerosis. HUMAN MOLECULAR GENETICS. 1999 Feb;8(2):157–64.

5. Garcia ML, Singleton AB, Hernandez D, Ward CM, Evey C, Sapp PA, et al. Mutations in neurofilament genes are not a significant primary cause of non-SOD1-mediated amyotrophic lateral sclerosis. Neurobiol Dis. 2006 Jan;21(1):102–9.

6. Daoud H, Valdmanis PN, Belzil V, Spiegelman D, Henrion E, Diallo O, et al. Resequencing of 29 candidate genes in patients with familial and sporadic amyotrophic lateral sclerosis. Arch Neurol. 2011;68(5):587–93.

7. Couthouis J, Raphael AR, Daneshjou R, Gitler AD. Targeted exon capture and sequencing in sporadic amyotrophic lateral sclerosis. PLoS Genet. 2014 Oct;10(10):e1004704.

8. Nakamura R, Sone J, Atsuta N, Tohnai G, Watanabe H, Yokoi D, et al. Next-generation sequencing of 28 ALS-related genes in a Japanese ALS cohort. Neurobiol Aging. 2016 Mar;39:219.e1-8.

9. Krüger S, Battke F, Sprecher A, Munz M, Synofzik M, Schöls L, et al. Rare Variants in Neurodegeneration Associated Genes Revealed by Targeted Panel Sequencing in a German ALS Cohort. Front Mol Neurosci. 2016 Oct 13;9:92.

10. Pang SYY, Hsu JS, Teo KC, Li Y, Kung MHW, Cheah KSE, et al. Burden of rare variants in ALS genes influences survival in familial and sporadic ALS. Neurobiol Aging. 2017 Oct;58:238.e9-238.e15.

11. Morgan S, Shatunov A, Sproviero W, Jones AR, Shoai M, Hughes D, et al. A comprehensive analysis of rare genetic variation in amyotrophic lateral sclerosis in the UK. Brain. 2017 Jun;140(6):1611–8.

12. Nishiyama A, Niihori T, Warita H, Izumi R, Akiyama T, Kato M, et al. Comprehensive targeted next-generation sequencing in Japanese familial amyotrophic lateral sclerosis. Neurobiology of Aging. 2017;53:194.e1-194.e8.

13. Garton FC, Benyamin B, Zhao Q, Liu Z, Gratten J, Henders AK, et al. Whole exome sequencing and DNA methylation analysis in a clinical amyotrophic lateral sclerosis cohort. Mol Genet Genomic Med. 2017 Jun 12;5(4):418–28.

14. Muller K, Brenner D, Weydt P, Meyer T, Grehl T, Petri S, et al. Comprehensive analysis of the mutation spectrum in 301 German ALS families. JOURNAL OF NEUROLOGY NEUROSURGERY AND PSYCHIATRY. 2018 Aug;89(8):817–27.

15. Zhang H, Cai W, Chen S, Liang J, Wang Z, Ren Y, et al. Screening for possible oligogenic pathogenesis in Chinese sporadic ALS patients. Amyotroph Lateral Scler Frontotemporal Degener. 2018 Aug;19(5–6):419–25.

16. Liu ZJ, Wei Q, Tao QQ, Liu GL, Ni W, Li HF, et al. Genetic spectrum and variability in Chinese patients with amyotrophic lateral sclerosis. Aging Dis. 2019;10(6):1199–206.

17. Tripolszki K, Gampawar P, Schmidt H, Nagy ZF, Nagy D, Klivényi P, et al. Comprehensive Genetic Analysis of a Hungarian Amyotrophic Lateral Sclerosis Cohort. Front Genet. 2019;10:732.

18. Chen W, Xie Y, Zheng M, Lin J, Huang P, Pei Z, et al. Clinical and genetic features of patients with amyotrophic lateral sclerosis in southern China. Eur J Neurol. 2020;27(6):1017–22.

19. Lin F, Lin W, Zhu C, Lin J, Huang H, Zhu J, et al. Sequencing of neurofilament genes identified NEFH Ser787Arg as a novel risk variant of sporadic amyotrophic lateral sclerosis in Chinese subjects. BMC Med Genomics. 2021;14(1):222.

20. Giguet-Valard AG, Bellance R, Jeannin S, Duclos S, Olive P, Allard-Saint-Albin O, et al. SOD1-related ALS with anticipation in a large family from Martinique. Amyotrophic Lateral Scler Frontotemporal Degener [Internet]. 2021;((Giguet-Valard, Bellance, Jeannin, Duclos, Olive, Allard-Saint-Albin) Neurological and Neuromuscular Rare disorders department, CERCA, University Hospital Center of Martinique, Martinique, France). Available from: http://www.tandfonline.com/loi/iafd20

21. Shepheard SR, Parker MD, Cooper-Knock J, Verber NS, Tuddenham L, Heath P, et al. Value of systematic genetic screening of patients with amyotrophic lateral sclerosis. J Neurol Neurosurg Psychiatry. 2021 May 1;92(5):510–8.

22. McCann E, Henden L, Fifita J, Zhang K, Grima N, Bauer D, et al. Evidence for polygenic and oligogenic basis of Australian sporadic amyotrophic lateral sclerosis. JOURNAL OF MEDICAL GENETICS. 2021 Feb;58(2):87–95.

23. Kruger S, Battke F, Sprecher A, Munz M, Synofzik M, Schols L, et al. Rare Variants in Neurodegeneration Associated Genes Revealed by Targeted Panel Sequencing in a German ALS Cohort. FRONTIERS IN MOLECULAR NEUROSCIENCE. 2016 Oct 13;9.
